# Metacercariae of *Posthodiplostomum* Dubois, 1936 (Diplostomidae) infecting freshwater fish worldwide: infection patterns, host-parasite interactions, distribution range, and disease control

**DOI:** 10.1590/S1984-29612026045

**Published:** 2026-10-09

**Authors:** Marcos Tavares-Dias

**Affiliations:** 1 Empresa Brasileira de Pesquisa Agropecuária – Embrapa Amapá, Macapá, AP, Brasil

**Keywords:** Diseases, fish, hosts, parasites, treatment, Trematoda, Doenças, peixes, hospedeiros, parasitos, tratamento, Trematoda

## Abstract

This study aimed to investigate the infection patterns, host-parasite interactions, distribution ranges, and control diseases by *Posthodiplostomum* metacercariae in freshwater fish on a global scale. We analyzed parasitological data from 203 freshwater fish species across 17 orders and 44 families, predominantly representing the Leuciscidae and Cichlidae families. The analysis identified a total of 10 *Posthodiplostomum* metacercariae species, although several studies did not identify the parasites to the species level. In host fish, prevalence, intensity and abundance levels of *Posthodiplostomum* metacercariae ranging from low to high. The metacercariae typically infected the body surface, musculature, mesentery or abdominal cavity, and internal organs of fish hosts, causing pathological effects that ranged from tissue damages to mortality, particularly in fry and juveniles. Regarding geographical distribution, metacercariae of *Posthodiplostomum minimum* occur across North America, whereas *Posthodiplostomum vancleavei*, *Posthodiplostomum ptychocheilus* and *Posthodiplostomum centrarchi* range from Canada to the United States. *Posthodiplostomum cuticola*, *P. centrarchi* and *Posthodiplostomum brevicaudatum* are distributed from Europa to Asia, while *Posthodiplostomum scardini* is restricted to Europe. In the Neotropics, metacercariae of *Posthodiplostomum nanum* occurs across South America, while *Posthodiplostomum macrocotyle* spans both South and Central America. Finally, this study highlights key research gaps particularly regarding parasite identification, prevention and treatments strategies.

## Resumo

Este estudo investigou os padrões de infecção, interações hospedeiro-parasito e distribuição geográfica e controle de doenças por metacercárias de *Posthodiplostomum* em peixes de água doce ao redor do mundo. Em 203 espécies de peixes abrangendo 17 ordens e 44 famílias houve uma predominância Leuciscidae e Cichlidae. Um total de 10 espécies de metacercárias de *Posthodiplostomum* foi identificada, haja vista que vários estudos não identificaram a espécie de parasito. Nos peixes, os níveis de prevalência, intensidade e abundância de metacercárias de *Posthodiplostomum* variam de baixos a altos. As metacercárias infectavam principalmente a superfície corporal, musculatura, mesentério ou cavidade abdominal e órgãos internos dos peixes, causando efeitos patológicos desde danos teciduais até a mortalidade, principalmente de alevinos e juvenis. Metacercárias de *Posthodiplostomum minimum* ocorrem por toda a América do Norte, enquanto *Posthodiplostomum vancleavei*, *Posthodiplostomum ptychocheilus* e *Posthodiplostomum centrarchi* ocorrem do Canadá aos Estados Unidos. *Posthodiplostomum cuticola*, *P. centrarchi* e *Posthodiplostomum brevicaudatum* tem distribuição da Europa à Ásia, enquanto *Posthodiplostomum scardini* está restrita à Europa. Na região Neotropical, metacercárias de *Posthodiplostomum nanum* ocorrem por toda a América do Sul, enquanto *Posthodiplostomum macrocotyle* está distribuído pela América do Sul e Central. Por fim, destacou-se lacunas importantes, principalmente quanto a identificação dos parasitos e às estratégias de prevenção e tratamento.

## Introduction

Metacercariae of trematodes are the most abundant helminths and frequently infest different tissues in fish. As part of the complex life cycles of parasites, these metacercariae serve as ecological bioindicators and may reflect the presence of different host taxa in ecosystems (Kudlai et al., 2026). *Posthodiplostomum* Dubois, 1936 are trematodes that the metacercariae with a wide global range within the Diplostomidae, a family of digeneans with a complex life cycle and a wide distribution around the world, as they infest various invertebrates and vertebrates, including fish (Hoffman, 1958; Pérez-Ponce de León et al., 2022; Chaudhary et al., 2023; Atkinson et al., 2023; Kudlai et al., 2026). Although around 40 species of *Posthodiplostomum* are currently known to be distributed globally (Achatz et al., 2025; WoRMS, 2026), some species have restricted range to certain regions (Cech et al., 2020). Metacercariae of *Posthodiplostomum minimum* (MacCallum, 1921) Dubois, 1936 particularly infect freshwater fishes across North and Central America and have been reported infecting more than 100 species of centrarchids from North America (Rossiter & Davidson, 2018) and another 109 fishes across from Mexico (Pérez-Ponce de León et al., 2022). However, over 25 species of *Posthodiplostomum* metacercariae have been identified in fish throughout the world. They have been mainly described in fish from the Americas and Europe, and less frequently in fish from Asia (Pérez-Ponce de León et al., 2022; Dinh-Hung et al., 2023); while the global range of the metacercarial species associated with freshwater fish has not been fully explored. Furthermore, due to revisions of the genus *Posthodiplostomum* and related genera, the genera *Ornithodiplostomum* Dubois, 1936 and *Mesoophorodiplostomum* Dubois, 1936 underwent taxonomic reorganization and were synonymized with *Posthodiplostomum* (Achatz et al*.*, 2021; Díaz-Pernett et al., 2022; WoRMS, 2026). Recently, Burris-Otero et al. (2026) reported that *Posthodiplostomum nanum* Dubois, 1937 is likely a synonym of *P. minimum* in North America.

Adult stages of *Posthodiplostomum* species are found particularly inside the intestines of piscivorous birds (Pérez-Ponce de León et al 2022; González-García et al., 2024; Achatz et al., 2025; Bispo et al., 2025), which are the definite hosts of these digeneans, whose life cycle often involves two intermediate hosts: gastropod mollusks (e.g., genus *Physella, Gundlachia, Physa*, *Lymnaea*, *Radix* and *Planorbis*) and fishes (Hoffman, 1958; Ritossa et al., 2013; López-Hernández et al., 2018; Velázquez-Urrieta & Pérez-Ponce de León 2021; Achatz et al., 2025; Bispo et al., 2025), as detailed in Figure 1. Nevertheless, recent studies have provided the first molecular evidence that *Posthodiplostomum cuticola* (von Nordmann, 1832) Dubois, 1936 can use also leeches as first intermediate hosts (Rutkauskaitè-Sucilienè et al., 2025). Consequently, high infection levels by metacercariae in fish population may indicate an elevated abundance or density of snails and piscivorous birds in the environment. Both are part of the complex life cycle of trematodes and thus can serve as ecological indicators reflecting the presence of these hosts in environment (Chapman et al., 2015; Honnananda et al., 2024; Malloy et al., 2024; Hnilička et al., 2025; Kudlai et al., 2026). Interestingly, studies found that when *Rutilus rutilus* (Linnaeus, 1758), *Scardinius erythrophthalmus* (Linnaeus, 1758) and *Perca fluviatilis* Linnaeus, 1758, all fish that acted usually as the secondary intermediate hosts of *P. cuticola* metacercariae replaced *Cyprinus carpio* Linnaeus, 1758, this new aquaculture practice led to a sharp decline in intensity of this digenean in both *Phalacrocorax carbo* and *Ardea cinerea*, which are the definite hosts. This was caused by the high dependence of these birds (definite hosts) on *C. carpio* in their diet, which was replaced by *R. rutilus*, *S. erythrophthalmus* and *P. fluviatilis*, which had low infection with these metacercariae (Heneberg & Sitko, 2026). Therefore, the nature and extent of the effects that the metacercariae exercises on its host also heavily depend on characteristics of the surrounding biotope, because availability plays a key role in how hosts manage their parasite loads.

**Figure 1:**
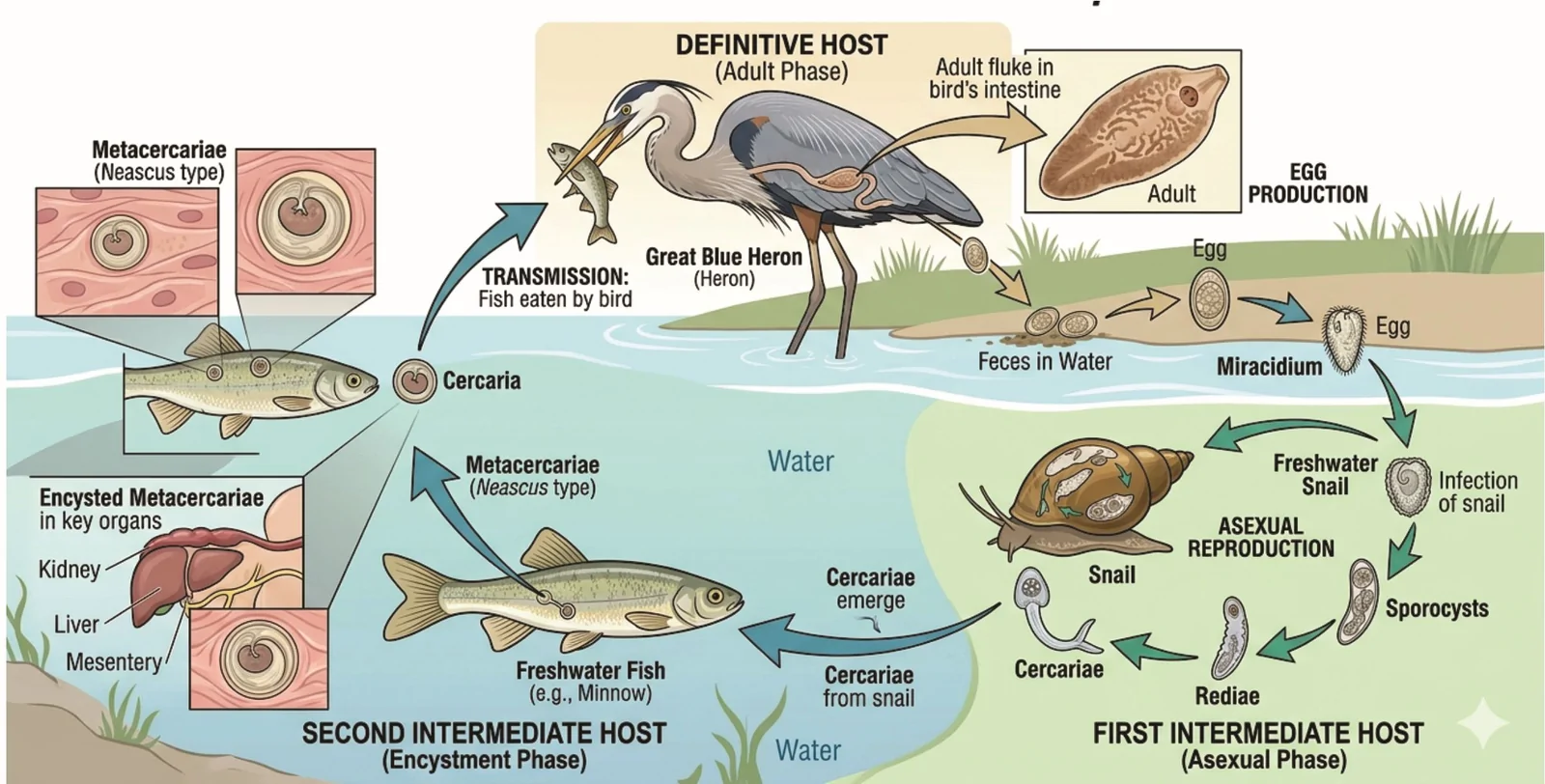
Life cycle of *Posthodiplostomum* spp. with stages within different hosts. The definitive hosts are fish-eating birds (mostly Ardeidae) in which the adult parasites live in their intestines and the eggs produced are released into aquatic environment via feces. In the first intermediate hosts, the eggs hatch and the larva infect freshwater snails (Mollusca: Gastropoda), and inside the snails the parasites reproduce asexually and develop into free-swimming cercariae. In second intermediate host, the cercariae emerge from the snail, swim, and actively penetrate the skin of fish hosts, and migrate to various organs and encyst as metacercariae. The transmission of larval parasites occurs when an infected fish is eaten by a suitable fish-eating bird, and the metacercariae are released, develop into adults in the bird's intestine, and begin the cycle again.

Note: This figure was created with the assistance of artificial intelligence tool (Gemini).

Identification of the metacercariae stages of *Posthodiplostomum* species has often been overlooked in many species of fish populations, because identification based solely on morphology is difficult. Furthermore, metacercariae sampling and assessment are time-consuming and laborious and require experts for accurate identification of species. With that in mind, molecular methods have emerged as an effective solution for detecting, characterizing, and monitoring parasitic infections. Recently, morphological and molecular studies resurrected and transferred the lineage of *Posthodiplostomum* metacercariae infecting Leuciscidae in North America to the earliest name, i.e., *Posthodiplostomum vancleavei* (Agersborg, 1926) Burris-Otero, Achatz, Marcogliese, Reyda, Colston & Locke, 2026 (Burris-Otero et al., 2026). However, this accurate identification is vital for enabling knowledge of biodiversity and the global range of species, as well as for disease surveillance, epidemiology (Kudlai et al., 2026), host-parasite interactions, and disease control. These metacercariae may have considerable impacts on fish, because their infection rates can be among the factors that affect host populations. Despite the growing number of studies on *Posthodiplostomum* metacercariae in fish populations, knowledge regarding histopathology caused by infections, and infection processes and patterns remains limited.

Cercariae of *Posthodiplostomum* are released from the snails and penetrate host fish, encysting as metacercariae into fish (Hoffman, 1958; Rossiter & Davidson, 2018; Dinh-Hung et al., 2023), causing the black spot disease or posthodiplostomatosis in different internal and external tissues of the hosts. In fish, black-spot disease begins in the skin and other tissues due to the deposition of melanocytes around metacercariae (Baharsefat et al., 1974; Hangan et al., 2013; Innal et al., 2020; Chaudhary et al., 2023) as shown in Figure 2. In general, *Posthodiplostomum* metacercariae encyst in fins, skin, subepidermal tissue, gills and fish muscles under the skin of hosts, and migration to other tissues occurs almost exclusively through muscles (Hoffman, 1958; Hendrickson, 1979; Conn et al., 2008; Karimian et al., 2013). This penetration and migration of the metacercariae can cause a decrease in fish body condition and growth, mechanical damages, hemorrhages, body deformation, muscular fibers necrosis, kidney and liver dystrophy, especially in fry, and are also able to contribute to hepatomegaly and splenomegaly (Markovic & Krsmanovic, 2008; Ondráčková et al., 2019; Innal et al., 2020; Chaudhary et al., 2023; Dinh-Hung et al., 2023; Honnananda et al., 2024). Consequently, *Posthodiplostomum* metacercariae can be pathogenic for both wild and farmed fish when severe infections occur that can lead to outbreaks in mortality, particularly with fingerlings and juveniles (Mitchell et al., 1982; Rossiter & Davidson, 2018; López-Hernández et al., 2018; Hamouda & Bazh, 2019; Cech et al., 2020; Dinh-Hung et al., 2023; Honnananda et al., 2024). These metacercariae are a major concern to both fisheries and aquaculture, given that high infection levels may cause give the fish an unattractive appearance that render them undesirable to both the fish processing industry and consumers. Furthermore, treatments for controlling infections by these metacercariae are difficult, especially when they invade the internal tissues of host fish. Overall, metacercariae are environmentally and economically important as they can reduce fish production, health and growth, also leave them more vulnerable to other infections, and even result in mass fish mortality (Kudlai et al., 2026).

**Figure 2:**
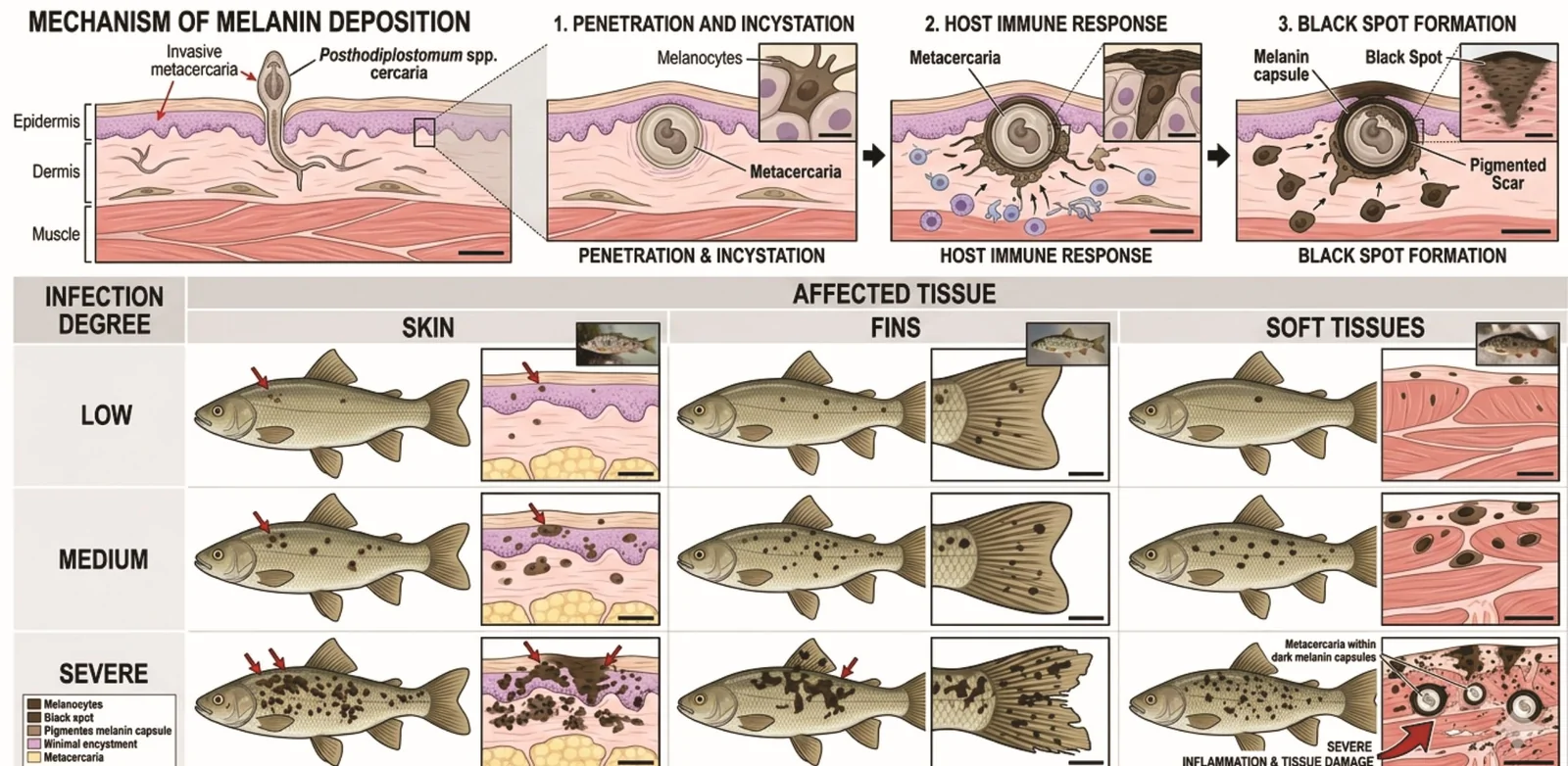
Simplified process of the formation of black spot disease in fish tissues caused by metacercariae of *Posthodiplostomum* species and mechanism of melanin deposition at the infection site.

Note: This figure was created with the assistance of artificial intelligence tool (Gemini).

Infections of *Posthodiplostomum* metacercariae occur in both wild and farmed fish populations .in many geographic regions (Athokpam & Tandon, 2014; Rossiter & Davidson, 2018; López-Hernández et al., 2018; Hamouda & Bazh, 2019; Innal et al., 2020; Díaz-Pernett et al., 2022; Kudlai et al., 2026) and depend on various abiotic and biotic factors (Ondráčková et al., 2004a; Zargar et al., 2013; Chapman et al., 2015; Gilbert & Avenant-Oldewage, 2017; Rossiter & Davidson, 2018; Honnananda et al., 2024; Hnilička et al., 2025). *Posthodiplostomum* metacercariae have become increasingly recognized as important bioindicators of environmental changes. These larval stages have been used to assess the health status of aquatic environments, because metacercariae infections often increase in fish populations in polluted environments (Gilbert & Avenant-Oldewage, 2017). Environments with pollution show increased nutrient runoff leading to eutrophication, which drives a high population of snails, the first intermediate hosts and attracts piscivorous birds. This results in a massive release of *Posthodiplostomum* cercariae which development into metacercariae that overwhelm host fish immune systems. Non-polluted environments show a balanced ecosystem with limited nutrients, fewer snail hosts, and fish with strong immune responses, resulting in minimal infections *Posthodiplostomum* metacercariae infection in fish (Ondračková et al., 2004a; Thilakaratne et al., 2007; Zargar et al., 2012; Chapman et al., 2015; Taglioretti et al., 2018; Lebedeva et al., 2020; Honnananda et al., 2024; Hnilička et al., 2025).

Although is well- known that parasites regulate host fish population density and can influence energy flow and nutrient cycling within aquatic food webs (Runghen et al., 2021; Côté et al., 2026), such interaction between fish and *Posthodiplostomum* metacercariae globally have not been explored. However, host-parasite interaction studies are crucial for monitoring ecosystem health, aquaculture and fisheries management and for understanding biodiversity, as well as for assisting in identification of invasive species threats, range shifts of parasites and disease spillover (Chapman et al., 2015; Kvach et al., 2017; Runghen et al., 2021; Côté et al., 2026). In addition, the relationships between species richness and abundance of *Posthodiplostomum* metacercariae and ecosystem functions have implications for conservation ecology and biodiversity. The richness and abundance of these larval parasites may contribute to ecosystem stability, primarily by reflecting structural complexity, regulating disease dynamics, and facilitating energy flow within food webs (Sures et al., 2023). In the case of strong ecological perturbations, there is a species turnover that leads to maximum ecosystem function and stability. Environment perturbations can significantly affect the host-parasite systems at different levels, meaning that the parasitized hosts often present a response pattern different from that of uninfected hosts (Sures et al., 2023). With that in mind this study aimed to investigate the infection patterns, host-parasite interactions, geographic range, and treatments to control diseases by *Posthodiplostomum* metacercariae in freshwater fish globally. These studies are also vital for mapping and improving knowledge of the dynamics of the different ecosystems. This knowledge may lead to more precise mapping of global ranges patterns of these larval trematodes in freshwater fish, thus enabling estimates of the parasite species and improving understanding of their infestation patterns in host fish species with wide geographic distributions, in addition to determining range limits of *Posthodiplostomum* species associated with these hosts.

## Material and Methods

### Literature search parameters

A review of *Posthodiplostomum* metacercariae species infesting freshwater teleost fish was conducted by searching the ISI, Redalyc, Google Scholar, Scopus, ScienceDirect, Web of Science, Zoological Record, CAB Abstracts, Lilacs, Scielo, Capes Periodicals, Aquatic Science, EBSCO, and Fisheries Abstract databases. The keywords used in the search were “*Posthodiplostomum* and freshwater fish.” The references listed in the articles were also checked to identify useful publications. Articles with questionable identification for both parasite species and fish species were not used in this study. The database for *Posthodiplostomum* metacercariae species associated with freshwater fish was compiled using taxonomic descriptions and surveys of these parasites’ occurrences published from 1940 to 2026. These data comprised surveys on metacercariae of species infecting farmed and wild fish distributed worldwide that yielded 506 samples of 260 articles selected.

The taxonomic nomenclature of the *Posthodiplostomum* species was based on WoRMS (2026), and the taxonomy of the host fish species was based on Froese & Pauly (2026). A total of 230 articles were selected and organized in a dataset that contained information on freshwater fish infected by *Posthodiplostomum* metacercariae species at a specific location and time. Some of the information from the samples included data on more than one host species. The data were organized into a data frame (extension “.txt”) with the following variables: (i) parasite species, (ii) infestation site, (iii) prevalence, (iv) mean intensity, (v) mean abundance, (vi) total of parasites recovered, as well as categorical factors: (i) host fish species, (ii) location of sample collection, (iii) family and order of host fish species, (iv) body length and weight, (v) correlation of intensity and abundance of parasites with body length and weight and (vi) type of infection distribution.

The ecological terms used (prevalence, intensity and abundance) were those recommended by Bush et al. (1997). To determine the interactions and infection sites of *Posthodiplostomum* metacercariae species, a bipartite package was used to construct a bipartite network. This analysis was performed using the R software package (R Development Core Team, 2026).

## Results

Our search yielded in a total of only 10 *Posthodiplostomum* metacercariae species, because around 35% of species have not been identified. These metacercariae species were *Posthodiplostomum brevicaudatum* (von Nordmann, 1832) Wisniewski, 1958; *Posthodiplostomum ptychocheilus* (Faust, 1917) Achatz et al., 2021; *Posthodiplostomum scardinii* (Shulman, 1952) Achatz et al., 2021; *Posthodiplostomum macrocotyle* Dubois, 1937; *Posthodiplostomum centrarchi* Hoffman, 1958; *Posthodiplostomum nanum* Dubois, 1937; *Posthodiplostomum biellipticum* Dubois, 1958; *P. minimum*; *P. vancleavei* and *P. cuticola*. Although our searches found *Posthodiplostomum* metacercariae infecting both farmed and wild fish with different feeding habits, these studies were concentrated predominantly in wild omnivorous fish populations. These metacercariae were found parasitizing 203 freshwater fish species from 17 orders, and 44 families, with a predominance of Leuciscidae and Cichlidae. Leuciscidae were found to have been infected by *P. brevicaudatum*, *P. ptychocheilus*, *P. scardinii*, *P. minimum*, *P. vancleavei*, *P. cuticola*, and species of *Posthodiplostomum* non-identified; while Cichlidae were infected by *P. macrocotyle*, *P. minimum*, *P. cuticola* and non-identified species of *Posthodiplostomum* (Table 1). However, *Lepomis macrochirus* Rafinesque, 1819 (Centrarchidae) was the most investigated host fish for metacercariae in these studies.

**Table 1:** Global geographic range of *Posthodiplostomum* metacercariae species associated with freshwater fishes of different feeding habits.

**Fish species**	**Feeding habit** *	**Family of fish**	**Order of fish**	**Parasite species** #	**Localities**
*Abramis brama*	Omnivorous	Leuciscidae	Cypriniformes	*Posthodiplostomum scardini*	Lithuania
*Abramis brama*	Omnivorous	Leuciscidae	Cypriniformes	*Posthodiplostomum cuticola*	Lithuania, Poland, Czech Republic and Russia
*Alburnoides bipunctatus*	Omnivorous	Leuciscidae	Cypriniformes	*Posthodiplostomum cuticola*	Lithuania
*Alburnus alburnus*	Omnivorous	Leuciscidae	Cypriniformes	*Posthodiplostomum cuticola*	Lithuania, Bulgaria and Czech Republic
*Ballerus ballerus*	Omnivorous	Leuciscidae	Cypriniformes	*Posthodiplostomum cuticola*	Czech Republic
*Blicca bjoerkna*	Omnivorous	Leuciscidae	Cypriniformes	*Posthodiplostomum cuticola*	Lithuania, Poland, Romania and Czech Republic
*Carassius auratus*	Omnivorous	Cyprinidae	Cypriniformes	*Posthodiplostomum cuticola*	Czech Republic
*Carassius carassius*	Omnivorous	Cyprinidae	Cypriniformes	*Posthodiplostomum cuticola*	Poland and Czech Republic
*Carassius gibelio*	Omnivorous	Cyprinidae	Cypriniformes	*Posthodiplostomum cuticola*	Lithuania, Bulgaria, Poland and Czech Republic
*Cobitis taenia*	Omnivorous	Cobitidae	Cypriniformes	*Posthodiplostomum cuticola*	Lithuania
*Ctenopharyngodon idella*	Herbivorous	Xenocyprididae	Cypriniformes	*Posthodiplostomum minimum*	Serbia, Ukraine and Czech Republic
*Cyprinus carpio*	Herbivorous	Cyprinidae	Cypriniformes	*Posthodiplostomum cuticola*	Hungary, Ukraine and Czech Republic
*Esox lucius*	Piscivorous	Esocidae	Esociformes	*Posthodiplostomum brevicaudatum*	Lithuania and Russia
*Gasterosteus aculeatus*	Carnivorous	Gasterosteidae	Perciformes	*Posthodiplostomum brevicaudatum*	Bulgaria
*Gobio gobio*	Omnivorous	Goodeidae	Cyprinodontiformes	*Posthodiplostomum cuticola*	Croatia
Hybrid *Chondrostoma nasus*	Omnivorous	-	-	*Posthodiplostomum cuticola*	Czech Republic
*Hypophtalmichthys molitrix*	Herbivorous	Xenocyprididae	Cypriniformes	*Posthodiplostomum cuticola*	Romania and Ukraine
*Hypophthalmichthys nobilis*	Herbivorous	Xenocyprididae	Cypriniformes	*Posthodiplostomum cuticola*	Serbia and Ukraine
*Leuciscus aspius*	Omnivorous	Leuciscidae	Cypriniformes	*Posthodiplostomum cuticola*	Czech Republic and Lithuania
*Leuciscus idus*	Omnivorous	Leuciscidae	Cypriniformes	*Posthodiplostomum cuticola* and *Posthodiplostomum scardini*	Czech Republic and Lithuania
*Leuciscus leuciscus*	Omnivorous	Leuciscidae	Cypriniformes	*Posthodiplostomum cuticola*	Lithuania
*Leucaspius delineatus*	Omnivorous	Leuciscidae	Cypriniformes	*Posthodiplostomum cuticola*	Czech Republic
*Lepomis gibbosus*	Carnivorous	Centrarchidae	Centrarchiformes	*Posthodiplostomum centrarchi*	Czech Republic, Bulgaria, Portugal and Germany
*Micropterus salmoides*	Carnivorous	Centrarchidae	Centrarchiformes	*Posthodiplostomum centrarchi*	Portugal and Ukraine
*Neogobius fluviatilis*	Omnivorous	Goodeidae	Gobiiformes	*Posthodiplostomum cuticola*	Lithuania
*Pelecus cultratus*	Carnivorous	Leuciscidae	Cypriniformes	*Posthodiplostomum cuticola*	Poland and Lithuania
*Perca fluviatilis*	Piscivorous	Percidae	Perciformes	*Posthodiplostomum cuticola* and *Posthodiplostomum brevicaudatum*	Lithuania, Poland, and Czech Republic
*Phoxinus dragarum*	Omnivorous	Leuciscidae	Cypriniformes	*Posthodiplostomum cuticola*	France
*Phoxinus phoxinus*	Omnivorous	Leuciscidae	Cypriniformes	*Posthodiplostomum cuticola*	France
*Phoxinus septimaniae*	Omnivorous	Leuciscidae	Cypriniformes	*Posthodiplostomum cuticola*	Spain
*Planiliza haematocheilus*	Omnivorous	Mugilidae	Mugiliformes	*Posthodiplostomum brevicaudatum*	Ukraine
*Proterorhinus semilunaris*	Omnivorous	Goodeidae	Gobiiformes	*Posthodiplostomum cuticola*	Russia
*Pseudorasbora parva*	Omnivorous	Goodeidae	Gobiiformes	*Posthodiplostomum cuticola*	Moldavia and Croatia
*Rhodeus amarus*	Omnivorous	Acheilognathidae	Cypriniformes	*Posthodiplostomum cuticola*	Czech Republic and Croatia
*Rhodeus sericeus*	Omnivorous	Acheilognathidae	Cypriniformes	*Posthodiplostomum cuticola*	Czech Republic
*Rutilus rutilus*	Omnivorous	Leuciscidae	Cypriniformes	*Posthodiplostomum cuticola*, *Posthodiplostomum brevicaudatum* and *Posthodiplostomum* sp.	Lithuania, Poland, Russia, Finland, Romania, Slovakia, Croatia and Czech Republic
*Scardinius erythrophthalmus*	Omnivorous	Leuciscidae	Cypriniformes	*Posthodiplostomum cuticola*, *Posthodiplostomum brevicaudatum* and *Posthodiplostomum scardini*	Lithuania, Poland, Romania and Czech Republic
*Salmo trutta*	Carnivorous	Salmonidae	Salmoniformes	*Posthodiplostomum cuticola*	Poland
*Squalius cephalus*	Carnivorous	Leuciscidae	Cypriniformes	*Posthodiplostomum cuticola* and *Posthodiplostomum scardini*	Lithuania, Serbia, Slovakia, Croatia and Czech Republic
*Alburnus escherichii*	Omnivorous	Leuciscidae	Cypriniformes	*Posthodiplostomum cuticola*	Turkey
*Alburnus filippii*	Omnivorous	Leuciscidae	Cypriniformes	*Posthodiplostomum cuticola*	Turkey
*Anabas testudineus*	Omnivorous	Anabantidae	Anabantiformes	*Posthodiplostomum* sp.	Vietnam
*Anatolichthys danfordii*	Carnivorous	Aphaniidae	Cyprinodontiformes	*Posthodiplostomum* sp.	Turkey
*Betta splendens*	Omnivorous	Osphronemidae	Anabantiformes	*Posthodiplostomum* sp.	Thailand
*Blicca bjoerkna*	Omnivorous	Leuciscidae	Cypriniformes	*Posthodiplostomum cuticola*	Turkey
*Capoeta tinca*	Omnivorous	Leuciscidae	Cypriniformes	*Posthodiplostomum cuticola*	Turkey
*Carassius auratus*	Omnivorous	Cyprinidae	Cypriniformes	*Posthodiplostomum cuticola*	Sri Lanka
*Cobitis taenia*	Omnivorous	Cobitidae	Cypriniformes	*Posthodiplostomum cuticola*	Turkey
*Cobitis turcica*	Omnivorous	Cobitidae	Cypriniformes	*Posthodiplostomum cuticola*	Turkey
*Carassius gibelio*	Omnivorous	Cyprinidae	Cypriniformes	*Posthodiplostomum cuticola* and *Posthodiplostomum* sp.	Uzbekistan and Siberia
*Channa argus*	Omnivorous	Channidae	Anabantiformes	*Posthodiplostomum* sp.	Japan
*Channa punctata*	Omnivorous	Channidae	Anabantiformes	*Posthodiplostomum* sp.	India
*Channa striata*	Omnivorous	Channidae	Anabantiformes	*Posthodiplostomum* sp.	Vietnam
*Cirrhinus mrigala*	Omnivorous	Cyprinidae	Cypriniformes	*Posthodiplostomum cuticola*	India
*Chondrostoma angorense*	Omnivorous	Leuciscidae	Cypriniformes	*Posthodiplostomum cuticola*	Turkey
*Chondrostoma colchicum*	Omnivorous	Leuciscidae	Cypriniformes	*Posthodiplostomum cuticola*	Turkey
*Cyprinus carpio*	Herbivorous	Cyprinidae	Cypriniformes	*Posthodiplostomum cuticola*	India
*Cyprinus carpio*	Herbivorous	Cyprinidae	Cypriniformes	*Posthodiplostomum brevicaudatum*	Uzbekistan
*Dermogenys pusilla*	Omnivorous	Zenarchopteridae	Beloniformes	*Posthodiplostomum* sp.	Thailand
*Labeo calbasu*	Omnivorous	Cyprinidae	Cypriniformes	*Posthodiplostomum cuticola*	India
*Labeo catla*	Omnivorous	Cyprinidae	Cypriniformes	*Posthodiplostomum cuticola* and *Posthodiplostomum* sp.	India
*Labeo rohita*	Omnivorous	Cyprinidae	Cypriniformes	*Posthodiplostomum cuticola*	India and Pakistan
*Lepomis gibbosus*	Carnivorous	Centrarchidae	Centrarchiformes	*Posthodiplostomum centrarchi*	Turkey
*Neogobius fluviatilis*	Omnivorous	Goodeidae	Gobiiformes	*Posthodiplostomum* sp.	Turkey
*Neogobius pallasi*	Omnivorous	Goodeidae	Gobiiformes	*Posthodiplostomum cuticola*	Iran
*Oreoleuciscus potanini*	Omnivorous	Leuciscidae	Cypriniformes	*Posthodiplostomum brevicaudatum*	Mongolia
*Perca fluviatilis*	Piscivorous	Percidae	Perciformes	*Posthodiplostomum* sp.	Russia (Siberia)
*Pethia conchonius*	Omnivorous	Cichlidae	Cichliformes	*Posthodiplostomum* sp.	India
*Proterorhinus marmoratus*	Omnivorous	Goodeidae	Gobiiformes	*Posthodiplostomum* sp.	Turkey
*Pseudaspius hakonensis*	Omnivorous	Leuciscidae	Cypriniformes	*Posthodiplostomum* sp.	Japan
*Rhynchocypris percnurus*	Omnivorous	Cyprinidae	Cypriniformes	*Posthodiplostomum* sp.	Russia (Siberia)
*Rutilus rutilus*	Omnivorous	Leuciscidae	Cypriniformes	*Posthodiplostomum cuticola* and *Posthodiplostomum* sp.	Russia (Siberia) and Kazakhstan
*Squalius cephalus*	Carnivorous	Leuciscidae	Cypriniformes	*Posthodiplostomum* sp.	Turkey
*Squalius pursakensis*	Carnivorous	Leuciscidae	Cypriniformes	*Posthodiplostomum cuticola*	Turkey
*Squalius turcicus*	Carnivorous	Leuciscidae	Cypriniformes	*Posthodiplostomum cuticola*	Turkey
*Schizopyge niger*	Herbivorous	Cyprinidae	Cypriniformes	*Posthodiplostomum* sp.	India
*Schizothorax esocinus*	Herbivorous	Cyprinidae	Cypriniformes	*Posthodiplostomum* sp.	India
*Trichopodus pectoralis*	Omnivorous	Osphronemidae	Anabantiformes	*Posthodiplostomum* sp.	Thailand
*Trichopodus trichopterus*	Omnivorous	Osphronemidae	Anabantiformes	*Posthodiplostomum* sp.	Vietnam
*Herichthys labridens*	Omnivorous	Cichlidae	Cichliformes	*Posthodiplostomum minimum* and *Posthodiplostomum* sp.	Mexico
*Hudsonius hudsonius*	Omnivorous	Leuciscidae	Cyprinodontiformes	*Posthodiplostomum vancleavei* and *Posthodiplostomum* sp.	United States and Canada
*Ictalurus punctatus*	Omnivorous	Ictaluridae	Siluriformes	*Posthodiplostomum minimum*	United States
*Lepomis auritus*	Carnivorous	Centrarchidae	Centrarchiformes	*Posthodiplostomum minimum* and *Posthodiplostomum centrarchi* North America	United States
*Lepomis cyanellus*	Carnivorous	Centrarchidae	Centrarchiformes	*Posthodiplostomum minimum,Posthodiplostomum centrarchid* and *Posthodiplostomum* sp.	United States
*Lepomis gibbosus*	Carnivorous	Centrarchidae	Centrarchiformes	*Posthodiplostomum minimum* and *Posthodiplostomum* sp.	United States and Canada
*Lepomis gulosus*	Carnivorous	Centrarchidae	Centrarchiformes	*Posthodiplostomum minimum* and *Posthodiplostomum* sp.	United States
*Lepomis humilis*	Carnivorous	Centrarchidae	Centrarchiformes	*Posthodiplostomum minimum* and *Posthodiplostomum* sp.	United States
*Lepomis macrochirus*	Carnivorous	Centrarchidae	Centrarchiformes	*Posthodiplostomum minimum*, *Posthodiplostomum centrarchi* and *Posthodiplostomum* sp.	United States and Canada
*Lepomis macrochirus* x *Lepomis cyanellus* hybrids	Carnivorous	-	-	*Posthodiplostomum minimum*	United States
*Lepomis marginatus*	Carnivorous	Centrarchidae	Centrarchiformes	*Posthodiplostomum minimum*	United States
*Lepomis megalotis*	Carnivorous	Centrarchidae	Centrarchiformes	*Posthodiplostomum minimum* and *Posthodiplostomum* sp.	United States
*Lepomis punctatus*	Carnivorous	Centrarchidae	Centrarchiformes	*Posthodiplostomum minimum*	United States
*Margariscus margarita*	Omnivorous	Leuciscidae	Cypriniformes	*Posthodiplostomum vancleavei*	Canada
*Maskaheros regani*	Omnivorous	Cichlidae	Cichliformes	*Posthodiplostomum* sp.	Mexico
*Mayaheros urophthalmus*	Omnivorous	Cichlidae	Cichliformes	*Posthodiplostomum minimum* and *Posthodiplostomum* sp.	Mexico
*Micropterus dolomieu*	Carnivorous	Centrarchidae	Centrarchiformes	*Posthodiplostomum minimum* and *Posthodiplostomum* sp.	United States and Canada
*Micropterus punctulatus*	Carnivorous	Centrarchidae	Centrarchiformes	*Posthodiplostomum minimum* and *Posthodiplostomum* sp.	United States
*Micropterus salmoides*	Carnivorous	Centrarchidae	Centrarchiformes	*Posthodiplostomum minimum* and *Posthodiplostomum* sp.	United States and Mexico
*Nocomis asper*	Omnivorous	Leuciscidae	Cypriniformes	*Posthodiplostomum vancleavei*	United States
*Noturus exilis*	Carnivorous	Ictaluridae	Siluriformes	*Posthodiplostomum minimum*	United States
*Oncorhynchus clarkii*	Carnivorous	Salmonidae	Salmoniformes	*Posthodiplostomum minimum*	United States
*Oreochromis* sp.	Omnivorous	Cichlidae	Cichliformes	*Posthodiplostomum minimum*	Mexico
*Paraneetroplus bulleri*	Omnivorous	Cichlidae	Cichliformes	*Posthodiplostomum* sp.	Mexico
*Paranotropis volucellus*	Omnivorous	Leuciscidae	Cypriniformes	*Posthodiplostomum* sp.	United States
*Perca flavescens*	Piscivorous	Percidae	Perciformes	*Posthodiplostomum* sp.	Canada
*Percopsis omiscomaycus*	Piscivorous	Percopsidae	Percopsiformes	*Posthodiplostomum minimum*	United States
*Petenia splendida*	Carnivorous	Cichlidae	Cichliformes	*Posthodiplostomum minimum*	Mexico
*Pimephales promelas*	Omnivorous	Leuciscidae	Cypriniformes	*Posthodiplostomum minimum*, *Posthodiplostomum vancleavei*, *Posthodiplostomum ptychocheilus* and *Posthodiplostomum* sp.	United States, Canada and Mexico
*Plagopterus argentlssimus*	Omnivorous	Leuciscidae	Cypriniformes	*Posthodiplostomum vancleavei*	United States
*Poecilia catemaconis*	Omnivorous	Poeciliidae	Cyprinodontiformes	*Posthodiplostomum* sp.	Mexico
*Poecilia formosa*	Omnivorous	Poeciliidae	Cyprinodontiformes	*Posthodiplostomum* sp.	Mexico
*Poecilia latipunctata*	Omnivorous	Poeciliidae	Cyprinodontiformes	*Posthodiplostomum* sp.	Mexico
*Poecilia mexicana*	Omnivorous	Poeciliidae	Cyprinodontiformes	*Posthodiplostomum minimum*	Mexico
*Poecilia reticulata*	Omnivorous	Poeciliidae	Cyprinodontiformes	*Posthodiplostomum minimum*	Mexico
*Poecilia sphenops*	Omnivorous	Poeciliidae	Cyprinodontiformes	*Posthodiplostomum minimum* and *Posthodiplostomum* sp.	Mexico
*Poeciliopsis gracilis*	Omnivorous	Poeciliidae	Cyprinodontiformes	*Posthodiplostomum* sp.	Mexico
*Poeciliopsis hnilickai*	Omnivorous	Poeciliidae	Cyprinodontiformes	*Posthodiplostomum minimum*	Mexico
*Poecilopsis infans*	Omnivorous	Poeciliidae	Cyprinodontiformes	*Posthodiplostomum* sp.	Mexico
*Pomoxis annularis*	Carnivorous	Centrarchidae	Centrarchiformes	*Posthodiplostomum minimum*	United States
*Pomoxis nigromaculatus*	Carnivorous	Centrarchidae	Centrarchiformes	*Posthodiplostomum* sp.	United States
*Profundulus oaxacae*	Omnivorous	Profundulidae	Cyprinodontiformes	*Posthodiplostomum* sp.	Mexico
*Profundulus punctatus*	Omnivorous	Profundulidae	Cyprinodontiformes	*Posthodiplostomum minimum* and *Posthodiplostomum* sp.	Mexico
*Pseudoxiphophorus bimaculatus*	Omnivorous	Poeciliidae	Cyprinodontiformes	*Posthodiplostomum* sp.	Mexico
*Pterophyllum scalare*	Omnivorous	Cichlidae	Cichliformes	*Posthodiplostomum* sp.	Brazil
*Pygocentrus nattereri*	Piscivorous	Serrasalmidae	Characiformes	*Posthodiplostomum* sp.	Brazil
*Rhinichthys osculus*	Omnivorous	Leuciscidae	Cypriniformes	*Posthodiplostomum* sp.	United States
*Rocio octofasciata*	Omnivorous	Cichlidae	Cichliformes	*Posthodiplostomum minimum* and *Posthodiplostomum* sp.	Mexico
*Sander vitreus*	Piscivorous	Percidae	Perciformes	*Posthodiplostomum minimum*	United States
*Skiffia lermae*	Omnivorous	Goodeidae	Gobiiformes	*Posthodiplostomum minimum* and *Posthodiplostomum* sp.	Mexico
*Thorichthys meeki*	Omnivorous	Cichlidae	Cichliformes	*Posthodiplostomum minimum* and *Posthodiplostomum* sp.	Mexico
*Trichromis salvini*	Omnivorous	Cichlidae	Cichliformes	*Posthodiplostomum minimum* and *Posthodiplostomum* sp.	Mexico
*Vieja fenestrata*	Omnivorous	Cichlidae	Cichliformes	*Posthodiplostomum* sp.	Mexico
*Vieja guttulata*	Omnivorous	Cichlidae	Cichliformes	*Posthodiplostomum* sp.	Mexico
*Xenotaenia resolanae*	Herbivorous	Goodeidae	Gobiiformes	*Posthodiplostomum minimum*	Mexico
*Xenotoca variata*	Carnivorous	Goodeidae	Gobiiformes	*Posthodiplostomum minimum*	Mexico
*Xiphophorus hellerii*	Omnivorous	Poeciliidae	Cyprinodontiformes	*Posthodiplostomum minimum*	Mexico
*Yuriria alta*	Omnivorous	Leuciscidae	Cypriniformes	*Posthodiplostomum minimum*	Mexico
*Amatitlania nigrofasciata*	Omnivorous	Cichlidae	Cichliformes	*Posthodiplostomum* sp.	Costa Rica
*Amatitlania siquia*	Omnivorous	Cichlidae	Cichliformes	*Posthodiplostomum* sp.	Costa Rica and Nicaragua
*Dajaus monticola*	Omnivorous	Mugilidae	Mugiliformes	*Posthodiplostomum* sp.	Puerto Rico
*Gobiomorus maculatus*	Carnivorous	Eleotridae	Gobiiformes	*Posthodiplostomum* sp.	Costa Rica
*Lepomis auritus*	Carnivorous	Centrarchidae	Centrarchiformes	*Posthodiplostomum* sp.	Puerto Rico
*Lepomis macrochirus*	Carnivorous	Centrarchidae	Centrarchiformes	*Posthodiplostomum* sp.	Puerto Rico
*Micropterus salmoides*	Carnivorous	Centrarchidae	Centrarchiformes	*Posthodiplostomum* sp.	Puerto Rico
*Parachromis managuensis*	Carnivorous	Cichlidae	Cichliformes	*Posthodiplostomum macrocotyle*	Puerto Rico
*Poecilia reticulata*	Omnivorous	Poeciliidae	Cyprinodontiformes	*Posthodiplostomum* sp.	Puerto Rico
*Andinoacara pulcher*	Omnivorous	Cichlidae	Cichliformes	*Posthodiplostomum* sp.	Venezuela
*Argolebias guarani*	Carnivorous	Rivulidae	Cyprinodontiformes	*Posthodiplostomum* sp.	Argentina
*Astronotus ocellatus*	Carnivorous	Cichlidae	Cichliformes	*Posthodiplostomum* sp.	Brazil
*Astronotus crassipinnis*	Carnivorous	Cichlidae	Cichliformes	*Posthodiplostomum* sp.	Brazil
*Astyanax* sp.	Omnivorous	Acestrorhamphidae	Characiformes	*Posthodiplostomum* sp.	Brazil
*Aequidens tetramerus*	Omnivorous	Cichlidae	Cichliformes	*Posthodiplostomum* sp.	Brazil
*Callichthys callichthys*	Omnivorous	Callichthyidae	Siluriformes	*Posthodiplostomum* sp.	Brazil
*Caquetaia kraussii*	Omnivorous	Cichlidae	Cichliformes	*Posthodiplostomum* sp.	Colombia
*Chaetobranchopsis orbicularis*	Omnivorous	Cichlidae	Cichliformes	*Posthodiplostomum* sp.	Brazil
*Chaetobranchus flavescens*	Omnivorous	Cichlidae	Cichliformes	*Posthodiplostomum* sp.	Brazil
*Cichlasoma bimaculatum*	Omnivorous	Cichlidae	Cichliformes	*Posthodiplostomum* sp.	Brazil
*Cichlassoma amazonarum*	Omnivorous	Cichlidae	Cichliformes	*Posthodiplostomum* sp.	Brazil
*Cnesterodon decemmaculatus*	Omnivorous	Poeciliidae	Cyprinodontiformes	*Posthodiplostomum nanum* and *Posthodiplostomum* sp.	Argentina
*Cnesterodon* sp.	Omnivorous	Poeciliidae	Cyprinodontiformes	*Posthodiplostomum* sp.	Argentina
*Curimata incompta*	Omnivorous	Curimatidae	Characiformes	*Posthodiplostomum* sp.	Brazil
*Galaxias maculatus*	Omnivorous	Galaxiidae	Galaxiiformes	*Posthodiplostomum* sp.	Argentina and Chile
*Geophagus brasiliensis*	Omnivorous	Cichlidae	Cichliformes	*Posthodiplostomum macrocotyle* and *Posthodiplostomum* sp.	Brazil
*Gymnotus carapo*	Piscivorous	Gymnotidae	Gymnotiformes	*Posthodiplostomum* sp.	Brazil
*Hemibrycon surinamensis*	Omnivorous	Stevardiidae	Characiformes	*Posthodiplostomum* sp.	Brazil
*Hoplerythrinus unitaeniatus*	Piscivorous	Erytrhinidae	Characiformes	*Posthodiplostomum* sp.	Brazil
*Hoplias malabaricus*	Piscivorous	Erytrhinidae	Characiformes	*Posthodiplostomum* sp.	Brazil
*Hoplosternum littorale*	Omnivorous	Callichthyidae	Siluriformes	*Posthodiplostomum* sp.	Venezuela
*Jenynsia lineata*	Omnivorous	Anablepidae	Cyprinodontiformes	*Posthodiplostomum nanum* and *Posthodiplostomum* sp.	Argentina
*Laetacara curviceps*	Omnivorous	Cichlidae	Cichliformes	*Posthodiplostomum* sp.	Brazil
*Lugubria strigata*	Carnivorous	Cichlidae	Cichliformes	*Posthodiplostomum* sp.	Brazil
*Mesonauta acora*	Omnivorous	Cichlidae	Cichliformes	*Posthodiplostomum* sp.	Brazil
*Mesonaut*a sp.	Omnivorous	Cichlidae	Cichliformes	*Posthodiplostomum* sp.	Brazil
*Myloplus rubripinnis*	Omnivorous	Serrasalmidae	Characiformes	*Posthodiplostomum* sp.	Brazil
*Poecilia reticulata*	Omnivorous	Poeciliidae	Cyprinodontiformes	*Posthodiplostomum nanum*	Brazil
*Satanoperca jurupari*	Omnivorous	Cichlidae	Cichliformes	*Posthodiplostomum* sp.	Brazil
*Trachelyopterus striatulus*	Omnivorous	Auchenipteridae	Siluriformes	*Posthodiplostomum macrocotyle*	Brazil
*Triportheus rotundatus*	Omnivorous	Triportheidae	Characiformes	*Posthodiplostomum* sp.	Brazil
*Bagrus bajad*	Carnivorous	Bagridae	Siluriformes	*Posthodiplostomum minimum*	Egypt
*Cyprinus carpio*	Herbivorous	Cyprinidae	Cypriniformes	*Posthodiplostomum* sp.	Ethiopia
*Enteromius radiatus*	Omnivorous	Cyprinidae	Cypriniformes	*Posthodiplostomum* sp.	South Africa
*Enteromius trimaculatus*	Omnivorous	Cyprinidae	Cypriniformes	*Posthodiplostomum* sp.	South Africa
*Garra dembecha*	Omnivorous	Cyprinidae	Cypriniformes	*Posthodiplostomum biellipticum*	Ethiopia
*Oreochromis niloticus*	Omnivorous	Cichlidae	Cichliformes	*Posthodiplostomum cuticola* and *Posthodiplostomum* sp.	Nigeria, Egypt and Ethiopia
*Tilapia sparrmanii*	Omnivorous	Cichlidae	Cichliformes	*Posthodiplostomum* sp.	South Africa

* According to Froese & Pauly (2026);

# Species of metacercariae were identified by molecular or morphological methods, and only few by both methods.

*Posthodiplostomum* metacercariae infected different organs of the same host and were recovered both in the fish body surface and muscles as well as in the mesentery, abdominal cavity and internal organs (Figure 3). The prevalence of *Posthodiplostomum* metacercariae ranged from low to high (0.005- 100%), and the intensity (0.007-1,685) and abundance (0.006- 1,685) also ranged from low to high (Figure 4). The total number of metacercariae recovered ranged from 2 to 22,881.The highest number of infections was caused by *P. minimum*, which infected a few fish. i.e., *L. macrochirus* and *Yuriria alta* (Jordan, 1880) (Leuciscidae).

**Figure 3:**
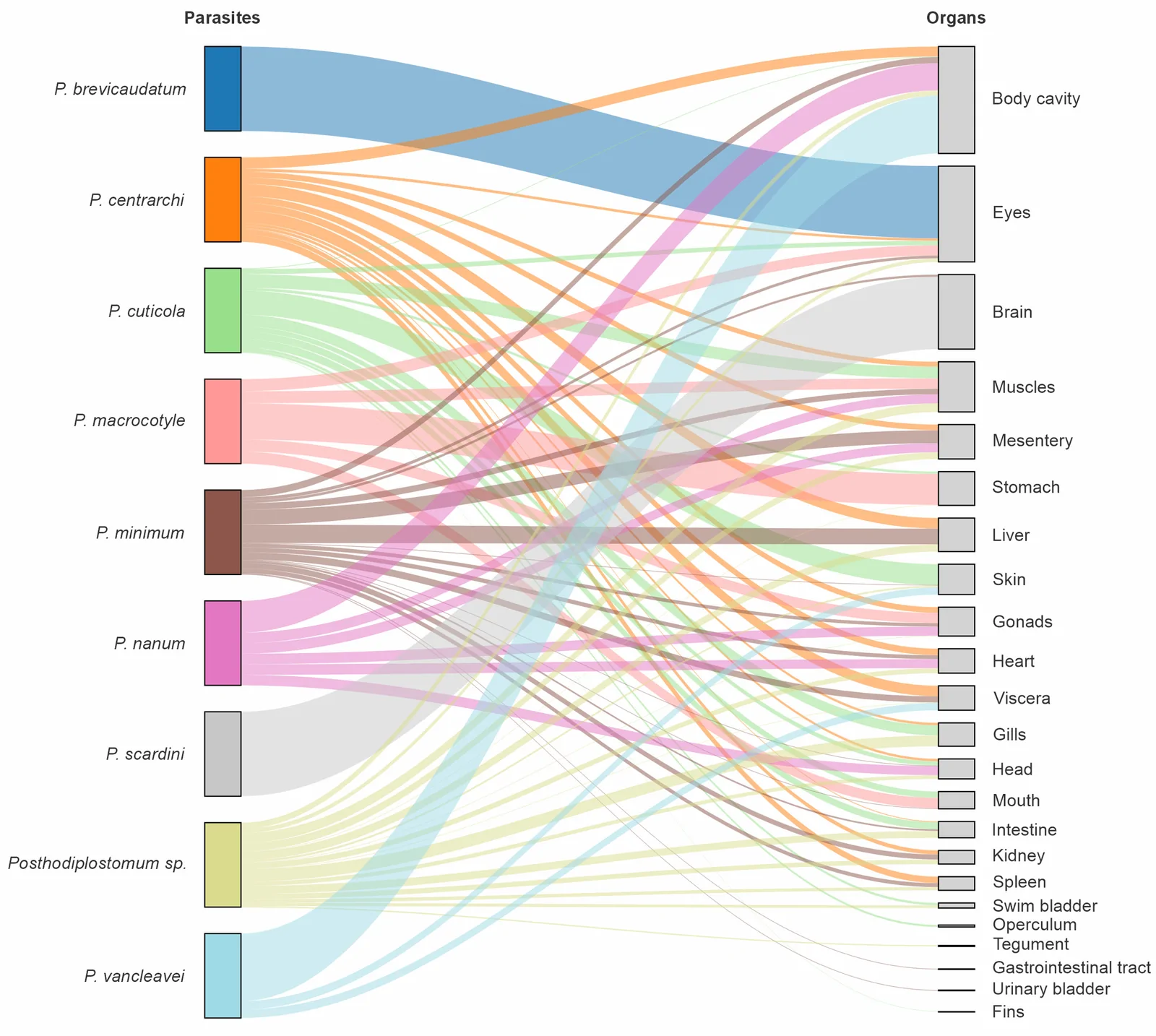
Bipartite network showing interactions between *Posthodiplostomum* metacercariae species in infection sites on freshwater fish.

**Figure 4:**
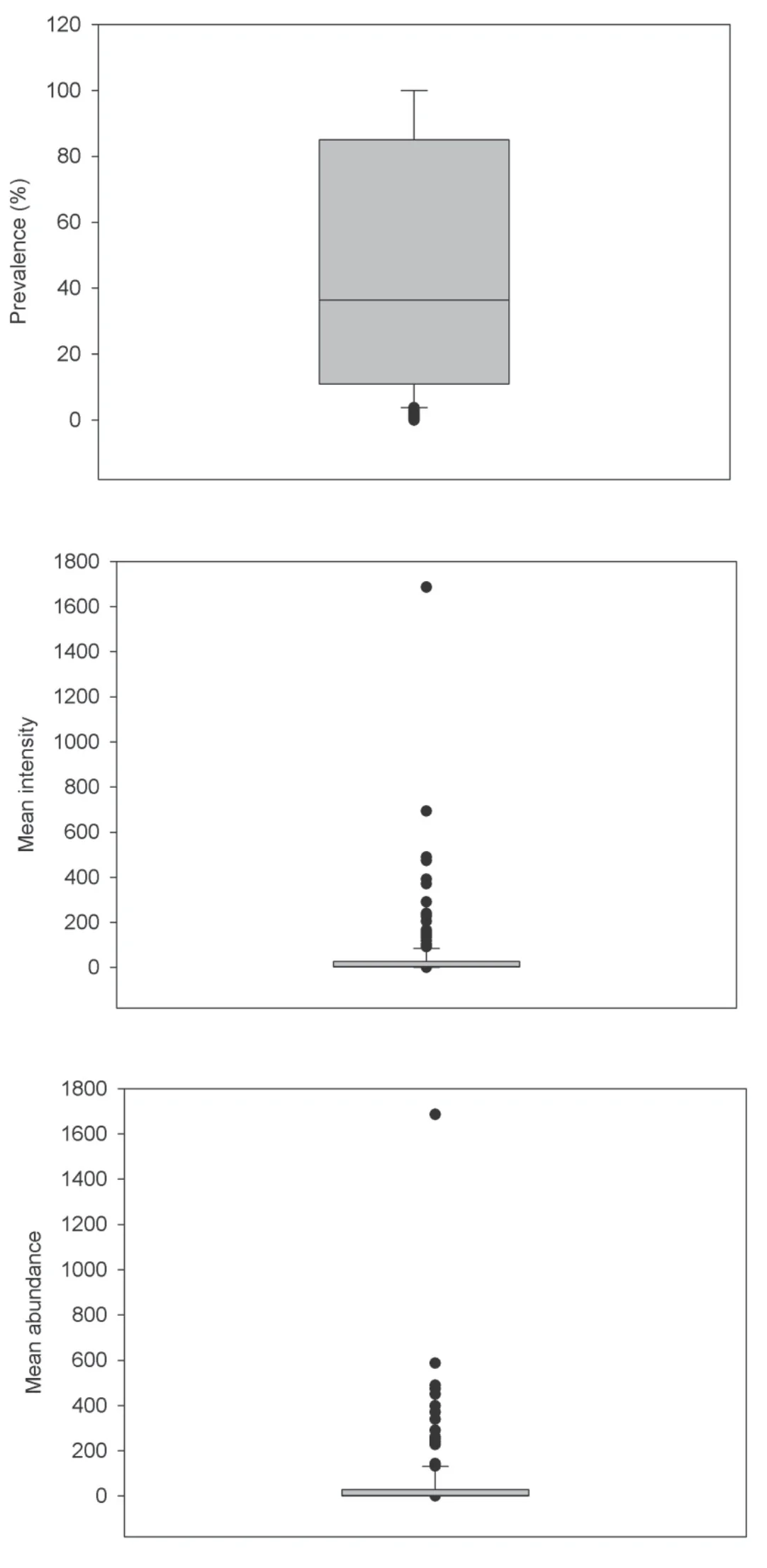
Quantitative descriptors of infection levels by global *Posthodiplostomum* metacercariae species in freshwater fish (Box plots represent medians, percentile ranges (25-75%), minimum-maximum and outlier values).

Metacercariae of *P. minimum* ranged throughout North America, from Canada to Mexico, while *P. vancleavei* and *P. ptychocheilus* metacercariae a ranged from Canada to the United States*.* Metacercariae of *P. cuticola*, *P. centrarchi* and *P. brevicaudatum* had ranges across countries from Europa to Asia, and *P. centrarchi* metacercariae has also been reported in fish, particularly throughout the United States, while *P. scardini* metacercariae have been identified infecting only few fish in Lithuania. *P. macrocotyle* Metacercariae have ranges both throughout Brazil and in Puerto Rico, while *P. nanum* metacercariae of have ranges throughout Argentina (Table 1).

## Discussion

### Host-parasite interactions and infection patterns of *Posthodiplostomum* metacercariae

We detected the following patterns within host fish and *Posthodiplostomum* metacercariae interactions: (a) both external and internal organs are microhabitats for infections; (b) aggregated dispersion of metacercariae infections; (c) prevalence varying from low to high, and mean intensity and abundance varying from low to moderate intensity, (d) positive correlation of intensity or abundance of metacercariae with the body size at the infracommunity level, and (e) majority of species identified only at genus level. The host-parasite dynamics in ecosystems are complex relationships between hosts and metacercariae of *Posthodiplostomum*, and thus highly influence their parasitism patterns in fish populations.

We found that few *Posthodiplostomum* metacercariae species have been identified to the species level, as with many identified only to the genus level due to various limitations. This difficulty stems from the limited morphological differentiation of these larvae, which are less developed than at their adult stages and often are found encysted. Furthermore, their morphology can vary depending on the fish host species and the specific microhabitat they occupy hosts (Moravec & Prouza, 2024; Kudlai et al., 2026), in addition to other factors. Consequently, identifying *Posthodiplostomum* metacercariae in fish populations requires integrative studies combining morphological and molecular analyses, as the molecular tools have proven to be an important and efficient approach for the taxonomic identification of these larval (Stoyanov et al., 2017; López-Hernández et al., 2018; Cech et al., 2020; Díaz-Pernett et al., 2022; Chaudhary et al., 2023; González-García et al., 2024; Kudlai et al., 2026). However, molecular analyses have not always achieved the expected success in resolving this issue. The main factors contributing to these failures in molecular tools are the high intraspecific variation, the presence of cryptic species complexes, gaps in GenBank databases, the selection of inadequate digenetic markers, host DNA contamination, and sample degradation (Kvach et al., 2017; Boone et al., 2018; Komatsu et al., 2020; Díaz-Pernett et al., 2022; Dinh-Hung et al., 2023; Velázquez-Urrieta et al., 2024; Bispo et al., 2025; Kudlai et al., 2026; Montes et al., 2026). It is suggested that a priority for future studies should be acquiring data from new molecular markers for type-species of genus *Posthodiplostomum*, i.e., *P. cuticula*, and for other species of the genus. Future analyses may also benefit from integrating other biological information, such as the hosts and the infection sites for metacercariae of this genus, because in this the type-species, the metacercariae form in the skin of fish hosts. However, there have been cases of controversial studies even when both morphological and molecular biological methods were used. Recently, Chaudhary et al. (2023) reported metacercariae inside livers of *Xenentodon cancila* Hamilton, 1822 that were morphologically similar to *Neascus pandei* Rai & Pande, 1964; while molecular biology studies confirmed these parasites as a *Posthodiplostomum* species identified as *Posthodiplostomum pandei*. Nevertheless, the description of *N. pandei* was provided by Pandey & Agrawal, 2013 (WoRMS, 2026). Posteriorly, Vankara et al. (2025) studying the morphology and histopathology of metacercariae of *Neascus hepatica* Chakrabarti, 1970 infecting the liver of *X. cancila* noted that these metacercariae of *P. pandei* described by Chaudhary et al. (2023) had body shapes and sizes closer to the metacercariae of *N. hepatica*. Therefore, *P. pandei* metacercariae were misidentified, as this species has not also been accepted (WoRMS, 2026).

We found that studies on metacercariae of *Posthodiplostomum* infecting freshwater fish around the world have often been concentrated in small omnivorous hosts, which are also predators. This is likely the result of the greater ease involved in sampling smaller fish, which are distributed across different ecosystems. Trematode metacercariae have a complex life cycle requiring more than one host species and are usually transmitted trophically (Taglioretti et al., 2024; Kudlai et al., 2026). A similar transmission pattern is observed for *Posthodiplostomum* metacercariae in fish populations. Because fish communities consist of species highly adapted to their environments based on available food resources, the fish exhibit diverse feeding habits driven by varied diets and a degree of dietary plasticity. Consequently, infection rates vary among hosts, as both fish diets and the interactions between *Posthodiplostomum* metacercariae and predation differ. This variation is particularly associated with the quantity o of prey infected by these parasites that fish encounter and ingest in their environment (Ondráčková et al., 2006; Mota-Júnior et al., 2024).

Host specificity, a measure of the diversity of hosts that a parasite can infect, is low when the parasite can infect many host fish, which indicates that it has no host specificity. It can be high when the parasite infects only a few host fish species, because it has an elevated host specificity. Digeneans are parasites that often display higher specificity for first-intermediate hosts (gastropod mollusks) than definitive hosts (birds) (Taglioretti et al., 2024) and secondary hosts (fish). Studies by Díaz-Pernett et al. (2022), based on molecular biology of *Posthodiplostomum* metacercariae, hypothesized that most species are restricted to a narrow range of host fish, e.g., infecting a single family. Nevertheless, *P. minimum* metacercariae of do not display host specify, because in addition to often parasitizing centrarchids, they have also been reported parasitizing species of numerous other fish families (Colley & Olson, 1963; Palmieri, 1976; Rossiter & Davidson, 2018; Pérez-Ponce de León et al., 2022; Burris-Otero et al., 2026), while *P. vancleavei* parasitize Leuciscidae species (Burris-Otero et al., 2026). Consequently, Lane et al. (2015) reported that the prevalence of *P. minimum* metacercariae was higher in *Lepomis* species than other Centrarchidae outside this genus, because such metacercariae are specialized in species of this genus.

Understanding complex patterns interactions between metacercariae of *Posthodiplostomum* and freshwater fish can provide vital data for fisheries and aquaculture, since they highlight the role of parasites and fish in ecosystems, in addition to providing information on host fish environments. Infection by *Posthodiplostomum* metacercariae species usually depends usually on multiple interacting factors rather than a single determinant, thus resulting in heterogeneous parasitic patterns among host fish populations. These factors have often been related to water quality, presence and density of snails (the first intermediate hosts), cercariae and piscivorous birds (definitive hosts) in environment, differences in spatial distribution of fish in different habitats, and differences in fish age, diet and immune resistance (Fischer & Kelso, 1990; Bhuthimethee et al., 2005; Buck & Lutterschmidt, 2017; Rossiter & Davidson, 2018; Lebedeva et al., 2020; Dinh-Hung et al., 2023; Yesuf et al., 2023; Honnananda et al., 2024; Malloy et al., 2024; Hnilička et al., 2025). Furthermore, these differences have also been linked to whether or not the fish species is native or introduced to ecosystems, because non-native fishes typically harbor fewer species of parasites than native fishes (Linder et al., 2012; Garrido-Olvera et al., 2017; Díaz-Pernett et al., 2022; Almeida et al., 2024). However, other factors have also been linked as influencing parasitism levels by *Posthodiplostomum* spp. metacercariae among host fish populations and should also be highlighted.

Fish body size is a biological measure particularly related to age of hosts, and has been a determinant factor in parasitic load, partly explaining variations found in fish populations. Host fish size is often a factor influencing *Posthodiplostomum* metacercariae recruitment, where larger hosts not only represent more pronounced targets for these stages, but are also frequently older, indicating increased exposure time to metacercariae (Atkinson et al., 2023). In *Micropterus salmoides* (Lacepède, 1802), the increase of *P. minimum* intensity with growth in length was related to increases in their body biomass (Fischer & Kelso, 1990). In *Lepomis gibbosus* (Linnaeus, 1758), the prevalence of *P. minimum* metacercariae was lower in younger hosts, increasing with growth in host ages (Cone & Anderson, 1977). In *R. rutilus*, *Blicca bjoerkna* (Linnaeus, 1758) and *Abramis brama* (Linnaeus, 1758), the abundance of *P. cuticola* increased with host length (Mierzejewska et al., 2004). Studies reported that *Jenynsia lineata* (Jenyns, 1842) was the host most infected by *P. nanum* metacercariae, even more than *Cnesterodon decemmaculatus* (Jenyns, 1842). Additionally, in *J. lineata*, the prevalence of was higher for smaller hosts, while the intensity was higher for hosts larger (Doma & Ostrowski de Núñez, 1994). However, the body size of *Lepomis cyanellus* Rafinesque, 1819 and *Lepomis macrochirus* Rafinesque, 1819 explained around 10% and 24% of abundance variation by *P. minimum* metacercariae, respectively (Fellis & Esch, 2004). In the eyes of *Gambusia affinis* (Baird & Girard, 1853), there is a positive correlation between the metacercariae of *Posthodiplostomum* sp. intensity and fish host size has been reported, because metacercariae were found in high intensities in all fish sampled (Griffin et al., 2017). In *L. macrochirus*, *L. cyanellus*, *M. salmoides* and *Lepomis auritus* (Linnaeus, 1758) the intensity of *P. centrarchi* metacercariae increased with growth in length of hosts (Malloy et al., 2024). Similarly, the abundance of *Posthodiplostomum* sp. metacercariae in *Lugubria strigata* (Günther, 1862) was positively correlated with host length, but not body weight (Mota-Júnior et al., 2024). It has suggested that the increased growth of fish parasitized by metacercariae is caused by parasite-induced foraging behavior, a parasite strategy to facilitate their transmission between hosts (Ondráčková et al., 2004b; Ondráčková et al., 2006).

As the age of *Neogobius pallasi* (Berg, 1916) increases, the *P. cuticola* metacercariae intensity and abundance infection also increases. This increase of the infection level in older hosts was due to larger size of the fish body surface area and long duration of parasite exposure (Karimian et al., 2013). In contrast, the prevalence of *P. coticola* in *Cobitis turcica* Hankó, 1925 tended to decrease with age of hosts, with a higher mortality rate in younger hosts. This mortality seems to have the most important impact of metacercariae infections for this fish population. Therefore, since the presence of these parasites has a major influence on the longevity of the fish, this parasitosis should be considered carefully (Kirankaya & Ekmekçi, 2011).

In *L. gibbosus*, the infection levels of *P. minimum* metacercariae are higher in male fish than in females (Cone & Anderson, 1977). Although *C. turcica* male populations are more abundant than females, the prevalence of *P. cuticola* in males was lower than in females (Kirankaya & Ekmekçi, 2011). On the other hand, in *N. pallasi*, infection levels by *P. cuticola* metacercariae were not influenced by the sex of hosts (Karimian et al., 2013).

The presence and success of a species of *Posthodiplostomum* metacercariae greatly depends on the complexity of biotic and abiotic factors acting on habitat and host fish. Among factors, seasonality can influence and limit the distribution of these metacercariae in fishes of certain regions. In *J. lineata* and *C. decemmaculatus* in Argentina, metacercariae infections in *P. nanum* indicated a recruitment during autumn/spring season and the no recruitment-period during winter/summer season (Doma & Ostrowski de Núñez, 1994). In *Phoxinus septimaniae* Kottelat, 2007 in Spain, the abundance of metacercariae of *P. cuticula* was higher in the spring (Almeida et al., 2024). In contrast, in *A. brama* examined in Russia, infections by *P. cuticola* metacercariae occurred during all seasons, while the prevalence was highest during the summer. Although the maximum life expectancy of *P. cuticola* metacercariae is about 3.5 years, live metacercariae were not found during the winter period, because water temperature was the main factor influencing the seasonality (Shukhgalter & Chukalova, 2002). Studies by Colley & Olson (1963) suggested that optimum temperatures for *P. minimum* metacercariae was close to those of host fish, varying from 10 to 29^o^C. Experimental studies of the life cycle of *P. brevicaudatum* reported that this cycle can be completed in 80 days under favorable conditions but may be delayed for as long as 4 years depending on ambient temperature. However, inside host fish the maximum life of metacercariae of *P. brevicaudatum* can last about 5 years, influenced by temperature (Chubb, 1979). The *Posthodiplostomum* metacercariae exhibit changes in their cholesterol levels, total lipid levels, and lipid composition that contrast with changes in tissue membrane adaptations of their host fish in response to environmental temperature variation. The metacercariae seem to rely on their host fish for adaptation within normal developmental temperature ranges, pooling both cholesterol and phosphatidylethanolamine as energy stores for their development and ontological transitions signaled by temperature (Welsh et al., 2006). In internal organs (i.e., intestine, urinary bladder, mesentery, stomach, liver, kidney and spleen) of *Bagrus bajad* (Fabricius, 1775) the occurrence of *P. minimum* metacercariae was highest during summer and lowest during winter (Hamouda & Bazh, 2019). In internal tissues of *Fundulus waccamensis* Hubbs & Raney, 1946 the higher prevalence and higher intensity of *P. minimum* metacercariae were in the summer and winter seasons (Burge & King, 2015). In *Pomoxis annularis* Rafinesque, 1818 in a lake in the USA, the number of *P. minimum* metacercariae was greater during summer than in the other seasons, coinciding with the increase in numbers of infected snails and the development and release of cercariae (Spall & Summerfelt, 1969). Similarly, in *L. macrochirus* and *L. auritus* collected from an urban watershed located in Columbus, Georgia (USA) the intensity of *P. minimum* was higher in summer than winter (Anderson et al., 2015). In *L. macrochirus* Louisiana (USA), the prevalence of *P. minimum* infection varied seasonally according to the two years investigated, because in one year it was lower during the spring and summer while in other it was lower during the fall, winter, and spring (Steinauer & Font, 2003). In *L. macrochirus* in the Lower Mississippi River, the intensity of *P. minimum* metacercariae decreased after the flooding period (Fischer & Kelso, 1990). In the eyes of *Squalius cephalus* (Linnaeus, 1758) in Turkey, infections by *Posthodiplostomum* sp. metacercariae were found only during autumn (Okkay & Özer, 2023). The prevalence and mean abundance of *P. cuticola* were higher in *R. rutilus* than in *S. erythrophthalmus* or *B. bjoerkna* in the Czech Republic, with both parameters showing maximum values in autumn and overwintering effect was detected on the *P. cuticola* infection (Ondráčková et al., 2004b). In *Galaxias maculatus* (Jenyns, 1842) of Patagua Lake, in Argentina, the prevalence of *Posthodiplostomum* sp. metacercariae was higher during summer than during spring (Ritossa et al., 2014).

In both *L. cyanellus* and *L. macrochirus* in North Carolina (USA), few seasonal variations by *P. minimum* metacercariae were attributed to a drop in abundances from March to April but remained constant throughout the rest of the year. Infections by such metacercariae are long-lived, and the only seasonal patterns likely to be detected would be a novel colonization or an accumulation over time (Fellis & Esch, 2004). In *Fundulus zebrinus* Jordan & Gilbert, 1883, infections by *P. minimum* metacercariae involved annual recruitment events. During extended periods of low water, intermediate host snail populations were higher while the prevalence of metacercariae remained at virtually 100% and number of parasites per host increased by an order of magnitude. During high-water events, both mean prevalence and abundance of parasites dropped, primarily because of failure of parasites to colonize new patches, with the resulting inclusion of young into populations (Janovy et al., 1997). In *C. turcica* from Pınarbaşi Springs (Turkey), during spring and autumn, the infection rates by *P. cuticola* were higher than during winter and summer, most probably due to the increasing density of migration of eating-fish birds into the region (Kirankaya & Ekmekçi, 2011). External and internal prevalence of *Posthodiplostomum* sp. metacercariae in *Anatolichthys danfordii* in Turkey were similar during the spring, summer and autumn, but were not found during winter. However, the intensity of metacercariae was lower in spring than in summer and autumn (Öztürk & Özer, 2019).

### Physiological and histomorphological alterations in fish infected by metacercariae of *Posthodiplostomum*

Histopathological studies on posthodiplostomatosis are crucial because they link tissue-level disease manifestation with physiological impairment, characterizing the severity of the pathology within fish population. These tissue analyses can also quantify host immunocompetence and chronic stress, which directly translate into reduced fitness, impaired locomotion, and high predation risks for fish, and decreases in feed conversion and growth rates. Furthermore, the life cycle of *Posthodiplostomum* species requires multiple hosts, these tissue alterations may serve as ecological biomarkers to monitor environmental health and multi-host population dynamics in aquatic ecosystems for such parasites. Nevertheless, studies on physiological and biochemical processes in fish populations infected by *Posthodiplostomum* metacercariae are very scarce. This scarcity occurs because posthodiplostomatosis is frequently accompanied by coinfections with other parasites rather than occurring in isolation.

The glycogen and protein contents of *R. rutilus* muscles infected by *P. cuticula* metacercariae were higher than in uninfected fish (Izvekova & Tyutin, 2011). In *L. macrochirus*, natural infection with *P. minimum* metacercariae directly altered serum protein levels. The immune system responded by producing antibodies, reflected in reduced albumin levels and increased beta and gamma-globulins, which are defense proteins (Meade & Harvey, 1969). In juvenile *R. rutilus* with muscular infection by different trematodes metacercariae, including *P. cuticola*, while the body length and weigh was lower than in uninfected fish, the glycogen content was higher (Izvekova & Zhokhov, 2016), which may be a result of a combination of several factors. One of them is probably the inhibited activity of the infected fish, because glycogen stores are spent rapidly during the active and dynamic short-term, especially in fish. These metacercariae can alter the behavioral patterns in host fish, given that penetration of these larvae into the fish leads to mechanical injuries of the muscles and thus significantly affects the movement activity of these hosts. Therefore, the chances that parasites will finish their life cycle lead to an increase in slow movements, which favors their predation by other hosts, causing a selective pressure. It is therefore not a result of parasitic pathogenicity. Hormonal changes can be another reason for these elevated glycogen concentrations in the infected fish muscles. Carbohydrates, which enter the fish organism as food, are stored in the muscles as glycogen, and insulin plays a primary role in this process. The high activity of hormones may result from excess glycogen storage in fish muscles (Izvekova & Tyutin, 2011). Thilakaratne et al. (2007) demonstrated that in *Notropis hudsonius* (Clinton, 1824), the numbers for the spleen and liver melanomacrophages centers presented a positive correlation with the abundance of *P. mimimum* metacercariae, whereas the total number of leukocytes had a positive correlation with the abundance of *P. ptychocheilus* metacercariae.

*Posthodiplostomum* metacercariae infections cause diverse histopathological alterations for both farmed and wild fish populations (Table 2). Nevertheless, histopathological studies on posthodiplostomatosis in fish populations are still quite limited (Hamouda & Bazh, 2019; Innal et al., 2020). Histological sections of the eye globe of infected *Anatolichthys danfordii* by species *Posthodiplostomum* showed metacercariae between the choroid and pigmented retina within the aqueous humor, and no metacercaria were detected in other locations in the eyes of fish (Öztürk & Özer, 2019). Similarly, histological sections of *G. affinis* showed *Posthodiplostomum* sp. metacercariae between the choroid and pigmented retina of both eyes that cause a disruption in the fish’s vision, and a resulting decrease in their fitness (Griffin et al., 2017). Specimens of *Alburnus escherichii* Steindachner, 1897, *Alburnus filippii* Kessler, 1877, *Capoeta tinca* (Heckel, 1843), *Chondrostoma angorense* Elvira, 1987, *Chondrostoma colchicum* Derjugin, 1899, *Squalius pursakensis* (Hankó, 1925) and *Squalius turcicus* De Filippi, 1865*,* severely infected by *P. cuticula* metacercariae exhibit multifocal black spots in all skin regions from mouth to caudal fin with differences in shape and diameter. In some fish, hyperemia was also observed around the lesions (Innal et al., 2020). In the edematous musculature of tilapia, encysted metacercariae, including *P. cuticola*, appeared surrounded with serous fluid containing a network of fibrin, which was attributed to irritation by parasites and their products (Eissa et al., 2011).

**Table 2:** Histomorphological alterations caused by species of *Posthodiplostomum* metacercariae in different tissues of the fish.

**Fish species**	**Parasite species**	**Skin and muscle alterations**	**References**
Non identified Cyprinidae	*Posthodiplotomum cuticola*	The cyst wall which surrounded metacercariae, composed of two distinct layers: the outer layer was very thick, and another consisted of dense fibrous connective tissue. Numerous melanoblasts containing large amounts of Melanin pigment which were localized in periphery of the cyst wall were found. Fibrous connective tissue that surrounded the cyst were elongated between the skeletal muscles as a narrow fibrous band and extended to next cyst. The inner layer was detached from the outer one and was quite free, but, in some instances a severe attachment was observed between two layers. The metacercariae were located centrally and was much smaller than the cyst, so there was a wide gap between parasite and cyst wall.	Baharsefat et al. (1974)
*Alburnus escherichii*, *Alburnus filippii*, *Capoeta tinca*, *Chondrostoma angorense*, *Chondrostoma colchicum*, *Squalius pursakensis* and *Squalius turcicus*	*Posthodiplotomum cuticola*	In skin of fish, the black spots were composed of metacercariae surrounded by thick fibrous capsules around the encysted parasites with periphery of the capsule containing numerous melanocytes; and most of the lesions contained one or more metacercariae of the metacercariae. The melanin capsules often present an opening on the surface of cuticle layer. Amount of the melanocytes was different in each nodule, either not observed at all or slight inflammatory reaction was found near the lesions. Melanocytes were also observed between the muscles near the cysts. The lesions were usually associated with migration of developmental stages of the parasite within the host tissues. Atrophy of the muscle near the parasitic lesion was commonly observed.	Innal et al. (2020)
*Scardinius erythrophthalmus*, *Rutilus rutilus* and *Blicca bjoerkna*	*Posthodiplotomum cuticola*	Musculocutaneous tissue showing presence of metacercaria in the central area of the cyst, which were located deep in tissues and surrounded by a connective tissue. This resulted in an aspect like a capsule in which numerous types of melanocyte cells, i.e., melanocytes and/or melanophages were seen. These cells had a dark brown pigment in the cytoplasm and a granular aspect masking the nucleus. On the periphery, slightly discreet, there was a cell infiltrate with few mononuclear and some heterophiles.	Negrea et al. (2015)
*Labeo catla* and *Pethia conchonius*	*Posthodiplotomum* sp.	The metacercariae-infected musculocutaneous tissues of host fish showed a proliferation of melanocytes just beneath the epidermis and a melanization of the fibrous capsule surrounding the metacercariae, which had pigmented areas along with slightly raised nodular structures. The surrounding muscular tissue suffered from medium to high levels of pressure atrophy and marked degeneration as well as focal necrosis. Infiltration cells such as mononuclear leukocytes that were of numerous immune observed in the fibrous capsule encircling the parasite. A melanization in the fibrous capsule surrounding the parasite was observed.	Baitha et al. (2021)
*Trichopodus pectoralis*	*Posthodiplotomum* sp.	Multiple encysted metacercariae were found just beneath the skin and in the subcutaneous musculature of the hosts, indicating a response by severe fibrosis. In the early stages of infection, the development of a cyst membrane, abundant fibroblasts, and no melanin deposits around the cyst were observed. As the infection advanced, the fibrosis capsule expanded, leading to a reduction in the number of fibroblasts, and the formation of a distinct cyst membrane became apparent. In the late stages of infection, a distinct shrinkage of the parasitic cyst membrane was observed, accompanied by a weak deposition of melanin within the fibrocytosis.	Dinh-Hung et al. (2023)
		**Liver alterations**	
*Lepomis macrochirus*	*Posthodiplostomum minimum*	Metacercariae penetration elicited hemorrhage at the site of invasion, congestion of adjacent venules, local edema, and an aggregation of phagocytic leucocytes at the point of entry. Double-triple membranes of the cyst wall, the erosion of hepatic tissue adjacent to cysts, disruption of outer cyst wall and vacuolation necrosis, and granulomatous hepatitis. In general, the large muscular oral sucker and acetabulum in the anterior body of parasite were clearly observed.	Amin & Elnaggar (2022)
*Bagrus bajad*	*Posthodiplostomum minimum*	The liver showed secondary degenerative changes, and with marked ballooning of hepatocytes with periportal hepatic necrosis	Hamouda & Bazh (2019)
*Pimephales promelas*	*Posthodiplostomum minimum*	Metacercariae stimulated a cellular inflammatory response, and some of them were encysted in tissues but appeared to be causing few damage to hepatocytes, and focal accumulations of macrophages were common in the tissues. Focal lesions in the process of repair, probably in areas where migration of metacercariae occurred, were occasionally seen, and such lesions contained fibroblasts which apparently produced collagenous connective tissue. Melanin-macrophage centers were diffused throughout the fibrinous exudate but were usually closely associated with encysted metacercariae	Mitchell et al. (1982)
*Chirostoma estor*	*Posthodiplostomum minimum*	In fish with moderate infections, which constituted the largest part of sample, the presence of encysted metacercariae and embedded in the hepatic parenchyma were observed. The cysts were constituted by a double wall, which an inner one of parasitic origin and the other outer one formed by the host; and a thick layer of fibrous connective tissue, made up mainly of fibroblasts and fibrocytes, and this fibrosis representing the final phase of leukocyte infiltration and granulation of the tissue. Atrophy of the hepatic parenchyma is observed, with collapsed hepatocytes due to the pressure exerted by the cysts. This manifests as a discordant sinusoidal organization, in contrast to the more peripheral hepatic sinusoids. Inflammatory cells, especially monocytes and lymphocytes with basophilic nuclei, were present. Eosinophilia, although moderate, was seen in the pericystic zones, as well as in some areas considered of migration into cercaria. Eosinophilia was associated with larval infections, and although this was not severe, this was well clear, especially in the regions near the parasite. In migration area of the cercariae occurred a peculiar arrangement of inflammatory cells and an incipient appearance of fibrous connective tissue, suggesting a pathway for parasite penetration. Also, was noted the presence of a well-defined granuloma characterized by the appearance of macrophages (epithelial cells and giant cells) surrounding a central area of ​​necrosis. At the edges of the macrophage zone, proliferation of fibroblasts was evident, among which lymphocytes and plasma cells were observed.	Osorio-Sarabia et al. (1986)
		**Spleen alterations**	
*Bagrus bajad*	*Posthodiplostomum minimum*	The spleen showed marked congestion of the hepatic sinusoids with depletion of both lymphoid elements and melanomacrophages cells.	Hamouda & Bazh (2019)
*Pimephales promelas*	*Posthodiplostomum minimum*	Presence of metacercariae stimulated a cellular inflammatory response, and some of them were encysted in spleen tissues	Mitchell et al. (1982)
		**Kidney alterations**	
*Bagrus bajad*	*Posthodiplostomum minimum*	Kidney demonstrated features of interstitial nephritis associated with degeneration and necrosis of the renal tubules and infiltration of inflammatory cells including eosinophilic granular cells, lymphocytes and macrophages.	Hamouda & Bazh (2019)
*Pimephales promelas*	*Posthodiplostomum minimum*	Presence of metacercariae stimulated a cellular inflammatory response, and some of them were encysted in kidney tissues	Mitchell et al. (1982)
		**Intestine alterations**	
*Bagrus bajad*	*Posthodiplostomum minimum*	The main histopathological alterations were the presence of different sized encysted metacercariae within the musculature of the infected intestine of hosts, particularly the subserosa layer. Sections of metacercarial cysts revealed that there were two layers, enclosed them as well as, they may contain more than one metacercariae in the same cyst. The cysts were also seen within tissue with deep necrotic changes which are mostly associated with inflammatory cells infiltration such as eosinophilic granular cells, lymphocytes and macrophages	Hamouda & Bazh (2019)
*Pimephales promelas*	*Posthodiplostomum minimum*	Presence of metacercariae stimulated a cellular inflammatory response, and some of them were encysted in the tissues	Mitchell et al. (1982)
		**Brain alterations**	
*Pimephales promelas*	*Posthodiplostomum ptychocheilus*	The outer cysts were composed of host fibroblasts which become anucleate and flatten around the metacercaria while the inner cysts were a dense non-cellular layer produced by secretions of the tegument. A thick filamentous glycocalyx, originating from the tegument, fills the space between the inner cyst and tegument. No morphologically identifiable collagen fibers or host leucocytes were identified in the outer cysts wall of metacercariae.	So & Wittrock (1982)

In histological sections of *L. gibbosus*, the *P. centrarchi* cysts were mostly found in the mesenteries and inside the interstitium of the kidney and liver, and less frequently in the muscle of the heart’s atrium. Conversely, they were never found in the skeletal muscles or in the ventricle muscle, but they readily adhered to the latter (Cech et al., 2020). Experimental trials with infection in fish with *P. ptychocheilus* cercariae showed that they reached the brain of the hosts within 24-48 h of penetration through the skin (Hendrickson, 1979; Conn et al., 2008), as complex development of metacercariae within the cranium of hosts involved the growth, encystment, and consolidation phases, each of which were affected by parasitic intensity (Sandland & Goater, 2000). Upon initial entry, these tended to occur deep within the brain and moved quickly to the outer edge of the optic lobes by day 2 post-infection (PI). By day 4 PI, the cercariae had increased markedly in size and somatic transformation that were accompanied by marked dissociation of host cells (Conn et al., 2008). In addition, these cercariae enter either the neural canal and spinal cord, or the brain via the spinal or cranial nerves. Their associated foramina, however, apparently remain in these peripheral nerves for only a short time, while in the cranial nerves they continue to the brain. Parasites becoming associated with spinal nerves traveling up the neural canal and/or spinal cord to the brain, and most arrive at the brain via the neural canal and spinal cord. When metacercariae developing within the brain occur in the optic lobes and cerebellum of host fish (Hendrickson, 1979) they can compromise their balance system and optomotor performance, affecting the host’s ability to detect and respond to prey, predators and conspecifics (Shirakashi & Goater, 2001), as well as other behaviors. Therefore, this increases the probability of fish infected by *Posthodiplostomum* metacercariae being consumed by predators (Ondráčková et al., 2006). Infections by *Posthodiplostomum* metacercariae, including encystment within the nervous system or in eyes of fish, are thus excellent examples of such parasites having the ability to alter the behavior of their hosts.

In *Pseudaspius hakonensis* (Günther, 1877), a few cysts containing live metacercariae adjacent to visceral organs were found, while others contained dead and degenerate metacercariae or were emptied and vestigial, suggesting that many parasites had been dead long before the fish were sampled. Cysts containing dead metacercariae or empty were all in the abdominal cavity, detached from visceral organs and surrounded by inflammatory cells of hosts (Komatsu et al., 2020). Infection by *P. minimum* metacercariae in visceral cavity of *Pimephales promelas* Rafinesque, 1820 also caused a highly cellular, fibrinous exudate the primary cell, with macrophages, eosinophils, and neutrophils also present in the tissue. Eosinophils were common in tissues of infected fish. Additionally, metacercariae were also found adjacent to the intestine, kidney, liver, and spleen (Mitchell et al., 1982).

*Lepomis macrochirus* coinfected by plerocercoids of *Proteocephalus* sp. and metacercariae of *P. minimum*, presented massive lesions turning virtually all abdominal viscera into a semi-solid aggregate adhering to the parietal peritoneum and kidneys. Parasite coinfection caused melanotic lesions in the liver and fibrosis ranging from scattered light and/or dark globular or tubular foci to numerous anastomosing cysts extending over large portions of the organ. Non-fibrotic livers carrying heavy loads of metacercariae but not plerocercoids presented little or no disruption of the normal arrangement and appearance of hepatocytes. Necrotic zones presented nuclear pyknosis, cell debris, isolated nuclei, extravasated erythrocytes and scattered leucocytes. Compressed hepatocyte zones were strongly basophilic with engorged capillaries and scattered fibroblasts, epithelioid cells and eosinophilic granulocytes. Liver parenchyma beyond the necrotic and compressed zones appeared normal. Parasitic fibrotic cysts were formed by concentric layers of dense non-cellular eosinophilic connective tissue infiltrated with granules and basophilic nuclear remnants. Most cysts surrounded a central space filled with amorphous basophilic material. Massive peritoneal cysts consisted of multilocular fibrous connective tissue which replaced much liver parenchyma and covered the liver and other visceral surfaces. Remains of dead plerocercoids and many metacercariae in fibrotic livers were also surrounded by concentric fibrous tissue; the surface of some metacercariae was invested with fibrous tissue, fibroblasts and leucocytes. Intrahepatic fibrotic cysts were surrounded by a granulomatous zone of densely packed fibroblasts, histiocytes and macrophages and/or epithelioid cells. Eosinophilic granulocytes were scattered in these zones, and parenchyma of fibrotic liver presented many macrophages and engorged blood vessels with thickened walls. Bile duct was externally thickened and fibrotic, and hepatic parenchyma in melanotic livers presented a marked increase in the frequency of melanomacrophages centers containing yellow and dark brown pigment granules. Dark pigment granules were diffusely scattered in the liver parenchyma (Mitchell et al., 1983).

### Geographic distribution of *Posthodiplostomum* metacercariae

*Posthodiplostomum minimum* metacercariae were the species with the widest range throughout North America, as they are main species found infesting freshwater fishes across Mexico, USA and Canada (Colley & Olson, 1963; Pérez-Ponce de León et al., 2022; Burris-Otero et al., 2026), and occasionally in Caribbean, South Asia and Africa (Hamouda & Bazh, 2019; Burris-Otero et al., 2026). However, in Central America, an area of an intense biotic exchange where the Nearctic and Neotropical biogeographical region converge, *P. minimum* metacercariae also infects fishes (Pérez-Ponce de León et al., 2022; Burris-Otero et al., 2026), while our findings show that studies on such metacercariae infesting fish throughout Central America were quite scarce.

In Europe only four species of *Posthodiplostomum* metacercariae are known, two being native species, i.e., *P. cuticola* and *P. brevicaudatum*, while *P. minimum* and *P. centrarchi* both originated from North America (Cech et al., 2020). Our results showed that *P. centrarchi* metacercariae of particularly ranges throughout the United States, while *P. scardini* metacercariae are restricted to Europe. However, the metacercariae of *P. cuticola*, *P. centrarchi* and *P. brevicaudatum* are found widely from Europa to Asia. It has been suggested that some species of *Posthodiplostomum* can have limited potential for infecting local fish fauna whereas others can have wider geographic range, because definitive host birds as Ardeidae (e.g. herons) can play an important role in dispersing these species as they seem to be causative agents of this dispersal between regions (Kvach et al., 2017, 2018). Furthermore, the translocation of fish may also have a major responsibility in the intercontinental species dispersion of these parasitic digeneans. For instance, *P. centrarchi*, of North American digeneans, seem to have been introduced in Europe together with fish from North America, i.e*.*, *L. gibbosus* and *M. salmoides* to some European countries, which later also commercialized these fish among other European countries, which thus spread this parasite across the continent (Kvach et al., 2017, 2018, 2024).

For South America, eight species of the genus *Posthodiplostomum* are currently listed by infect birds: *Posthodiplostomum grande* (Diesing, 1850), *Posthodiplostomum microsicya* (Dubois, 1937), *Posthodiplostomum obesum* (Lutz, 1928), *Posthodiplostomum giganteum* (Dubois, 1988), *Posthodiplostomum mignum* (Boero, Led & Brandetti, 1972), *Posthodiplostomum eurypygae* Achatz, Chermak, Bell, Fecchio & Tkach, 2021, *P. macrocotyle* and *P. nanum* (Gómez-Ruíz & Lenis, 2024). However, we found that only *P. nanum* and *P. macrocotyle* metacercariae of had spread widely in South America by infesting fish, while *P. macrocotyle* metacercariae of were present in fish from Puerto Rico, thus indicating that they also are widespread across Central America. Zhokhov (2012) reported metacercariae of *P. nanum* infesting fish from Ethiopia. However, integrative studies by Hoogendoorn et al. (2019) suggested that *P. nanum* metacercariae in freshwater fish across Africa must be considered *P. biellipticum* metacercariae of. Therefore, y *P. biellipticum* probably has a range restricted to Africa. *P. macrocotyle* Metacercariae of have ranges both throughout Brazil and Puerto Rico, while *P. nanum* metacercariae of is found throughout Argentina.

### Control and treatments for infections by *Posthodiplostomum* metacercariae

As aquaculture continues to expand, effective parasite control has become increasingly important, since the intensification of production systems promotes parasite proliferation and spread. Antiparasitic treatments are a major challenge for fish aquaculture, especially for controlling endoparasites. In addition to the limited number of antiparasitic drugs available, few are safe and effective for fish due to the difficulty in reaching the endoparasites inside the infected organs; and metacercariae of *Posthodiplostomum* are no exception. For control of *Posthodiplostomum* metacercariae infections only two chemotherapeutants have been evaluated, although both showed high effectiveness (Table 3). Although some biological control methods for reducing *Posthodiplostomum* metacercariae infections in aquaculture have also been recommended, available interventions for controlling this parasitosis remain limited and difficult. Control of metacercariae infections presents additional challenges, as they are larval stage parasites, which are often encysted in fish tissues.

**Table 3:** Effective treatments to control infections by *Posthodiplostomum* metacercariae in different fish.

**Chemotherapeutics**	**Dose**	**Method and time of treatment**	**Efficacy (%)**	**References**
Praziquantel	2 mg/L	Single bath for 24 h	100	Heckmann (2010)
Praziquantel	5 mg/kg/fish weight	Single dose via intramuscular or intraperitoneal inoculation	100	Heckmann (2010)
Praziquantel	56.7 mg/L	Single intramuscular injection	100	Heckmann (2010)
Praziquantel	150 mg/kg/fish weight	Diet for three days	100	Komatsu et al. (2020)
Praziquantel	200 mg/kg/fish weight	Single dose via oral	90.0	Yevtushenko (2019)
Praziquantel	5 mg/kg/fish weight	Single intramuscular injection	80	Bader et al. (2018)
Fenbendazole	0.6%	Diet for 14 days	100	Berezovsky et al. (2022)

Experimental studies with *P. minimum*, snails and *L. macrochirus* demonstrated that treatment by reduction of aquatic vegetation was not effective in controlling infections (Avault & Allison, 1965). However, in fish aquaculture, a reduction in the incidence of posthodiplostomatosis can be achieved by eliminating development of the cercariae snails in the ponds by disrupting the biological cycle of species of *Posthodiplostomum* metacercariae (Bader et al., 2017; Honnananda et al., 2024), as well as by expelling piscivorous birds in the fish farm area (Honnananda et al., 2024). Noatch & Whitledge (2011) suggested application of hydrated lime (31.7 kg/30.5 m of linear shoreline in a 1-m-wide swath) to reduce snail densities in fishponds, although the snail densities in several ponds had rebounded after 2 months. Soucek & Noblet (1998) reported that certain snail hosts (LC_50-7days_=38.0 mg/L) and free-swimming cercariae of *P. minimum* (LC_50-12h_=26.0 mg/L) were much more sensitive to total copper than were *L. macrochirus* hosts (LC_50-96h_= 0.892 mg/L). Sublethal concentrations based on these lethal concentrations (LC_50_) could be enough to affect interactions between host fishes and metacercariae and may eliminate both the infective form of the parasites and its first intermediate hosts. Exposure of *Physella acuta* snails Draparnaud, 1805, the first intermediate hosts for metacercariae of *P. mimimum*, to salinity of 7% significantly reduced their survival, indicating that salinity may be used in the management of these parasites in fish (Stockwell et al., 2011). Therefore, to avoid posthodiplostomatosis, preventive measures such as drying and mechanically cleaning ponds should be frequently implemented, because they are crucial management strategies for fish farms (Markovic et al., 2012). Additionally, to prevent metacercariae invading fishponds, it has also been recommended to fill them with water 10-12 days before stocking with fish. Thus, the cercariae that hatches from snails die without finding these first intermediate hosts and the risk of infection is reduced or disappears (Berezovsky et al., 2022).

In *P. hakonensis*, the mortality of metacercariae of *Posthotodiplostomum* sp. drastically increased 5 days after the termination of oral treatment with praziquantel (PZQ) and further increase one month later, although some remain alive. The presence of live metacercariae suggests that PZQ was not administered properly in some fish, because this chemotherapeutant presents a bitter taste (Komatsu et al., 2020). Metacercariae of *P. mimimum* inside liver and spleen of *M. salmoides* were inactivated by praziquantel. PZQ is readily absorbed and conveyed to the infected fish tissues through their cardiovascular system. After this treatment, the histological sections show outer membrane disruption and vacuolation of the parasites. When metacercariae of *P. mimimum* were exposed to water containing PZQ, after several hours the parasites became necrotic and disappeared (Heckmann, 2010). Conversely, in *Labeo calbasu*, (Hamilton, 1822), *Labeo catla* (Hamilton, 1822), *C. carpio* and *Cirrhinus mrigala* (Hamilton, 1822), treatments with calcium hydroxide (0.1 and 1.00 mg/L, calcium oxide and copper sulphate (20 mg/L), formalin (0.004 and 0.006 mg/L), gammexane (1-6-hexachlorocyclohexane) (0.2 and 5.0 mg/L), glacial acetic acid (0.001 mg/L), malachite green (0.01 mg/L), potassium permanganate (0.5, 1.0 and 100 mg/L) or sodium chloride (30 mg/L) were not effective against heavy infestations by *P. cuticola* metacercariae at the body surface. However, when treated with 0.03 mg/L of picric acid for 1.5 h. less heavily infected fish showed a decrease of infections by these ectoparasites (Singhal et al., 1986).

In the studies with antiparasitic treatments of fish, death of endoparasites usually is measured by loss of their movements; however, the challenge with metacercariae of *Posthodiplostomum* has been much greater. The death of these metacercariae that is also often measured as determined by their movement; although many encysted metacercariae have limited mobility and when they die, may take extended time to be removed by the host fish response. Therefore, it has been recommended not to assess the total number of metacercariae immediately following treatments but instead to use a method of propidium iodide staining for determining the number of metacercariae deaths (Bader et al., 2017, 2018, 2019). Additionally, it has been suggested that quantitative studies of metacercariae would benefit from this staining method to compare *Posthodiplostomum* with the relative level of antiparasitic treatment susceptibility or resistance of other species (Bader et al., 2019). Therefore, effective management relies on integrated strategies combining preventive husbandry, environmental manipulation, biological control, and targeted therapeutic interventions, with an emphasis on reducing infection to prevent the development of posthodiplostomatosis.

## Conclusions and Perspectives

Infection rates of *Posthodiplostomum* metacercariae across different host organs vary, as they are influenced by various factors. Although these infections are usually asymptomatic, although they may be deleterious, particularly for fingerlings or for cases of highly infected fish, since pathogenic species can cause histopathological changes and lethality. *Posthodiplostomum* metacercariae typically establish begin on the body surface of the host fish, because the cercariae usually penetrate the skin, before transforming into metacercaria that predominantly invade the skin and muscles, abdominal cavity, and sometimes other tissues. Infections in multiple organs of the same host may indicate a high parasitic intensity that results in lack of space in the primary microhabitat of the host, a biological flexibility of the species for dispersing into hosts, or stochastic event during migration route of metacercariae within the fish. The host-choice flexibility of some *Posthodiplostomum* metacercariae species appears to explain both the number of metacercariae infecting fish tissues and the development of these parasitic stages within the host.

Metacercariae of *P. minimum* and *P. cuticola* have no host specificity, while *P. brevicaudatum* metacercariae have a few host specificities, while metacercariae of *P. centrarchi* particularly infect Centrarchiformes. Metacercariae of *P. minimum*, *P. cuticola*, *P. centrarchi* and *P. brevicaudatum* were the species with the widest geographic ranges, while *P. vancleavei*, *P. scardini* and *P. macrocotyle* and *P. nanum* have restricted ranges. Despite the growing reports on infection by *Posthodiplostomum* metacercariae in fish globally, some gaps remain in the context of pathogenicity, and influence of aquaculture and fisheries practices on parasitism rates remains underexplored, hindering the development of targeted management strategies for aquaculture. Furthermore, future studies should use representative sampling, standardized diagnostic and reporting protocols, metacercariae species-level and molecular confirmation, and clear reporting of prevalence, intensity, abundance data.

The use of molecular methods is crucial for identifying *Posthodiplostomum* metacercariae species by enabling the accurate identification of species that would otherwise be difficult to distinguish using morphology alone. Furthermore, molecular tools may provide valuable insights into parasite diversity, phylogenetic relationships and population structure, which are crucial for developing effective prevention strategies and ensuring the health and sustainability of the farmed fish populations. Biological control measures may help reduce posthodiplostomatosis; however, treatment options for this disease are quite limited as they are currently dependent solely on praziquantel. Therefore, future studies should focus on other potential treatment products, as well as on eco-friendly interventions to improve prevention and management of this disease in intensive aquaculture systems. Preventive strategies that focus on environmental management, early detection, and host fish resilience are increasingly recognized as essential components of sustainable aquaculture. Finally, due to global climate change, one serious environmental problem that is a major concern, is the crucial need in this context to investigate the dynamic processes of development of infections by *Posthodiplostomum* metacercariae species in fish populations.

## Data Availability

Data will be made available upon request.
